# A critical review and classification of dementia risk assessment tools to inform dementia risk reduction

**DOI:** 10.1016/j.tjpad.2025.100333

**Published:** 2025-09-02

**Authors:** Md Hamidul Huque, Ranmalee Eramudugolla, Meiwei Li, Kim M. Kiely, Ruth Peters, Kaarin J. Anstey

**Affiliations:** a School of Psychology, University of New South Wales, High St, Kensington, NSW 2052, Australia; b Neuroscience Research Australia, 139 Barker St, Randwick, NSW 2031, Australia; c UNSW Ageing Futures Institute, University of NSW, High St, Kensington, NSW 2052, Australia; d School of Mathematics and Applied Statistics, and, School of Social Sciences, University of Wollongong, Northfields Ave, Wollongong NSW 2500, Australia; e School of Public Health, University of New South Wales, High St, Kensington, NSW 2052, Australia; f The George Institute of Global Health, 300 Barangaroo Ave, Barangaroo NSW 2000, Australia

**Keywords:** Dementia risk, Prevention, Prediction, Meta-analysis

## Abstract

Addressing modifiable dementia risk factors requires reliable risk assessment methods. We aimed to synthesise knowledge on risk scores for all cause dementia, Alzheimer’s disease (AD) and vascular dementia, classify them according to target population, evaluate their content, cost, appropriateness of validation studies, and suitability for implementing risk reduction guidelines. A systematic search was conducted of PubMed, Cochrane Collaboration, ProQuest, Scopus, Embase, and PsycINFO databases using a pre-registered protocol. Data on risk factors, target population, predictive validity, cost, and alignment with WHO guidelines were extracted. Random-effects meta-analysis was performed. Of 45 risk scores identified, 29 were for all-cause dementia, including 11 based on late-life cohorts, 6 on midlife, and 7 covering mid to late-life. The pooled C-statistic across development and validation studies of dementia risk scores was 0.69 (95 % CI: 0.67, 0.71). Development study AUCs were higher than validation study AUCs and dropped from 0.74 to 0.66 for risk scores developed for clinical samples and from 0.79 to 0.71 for AD specific scores (which include functional indicators non-independent of disease). There were no validated risk scores for vascular dementia. Dem-NCD, CogDrisk, ANU-ADRI and LIBRA risk scores incorporated most WHO-recommended risk factors and demonstrated accuracy comparable to the overall pooled C-statistic. We conclude that across the field, there are methodological limitations relating to validation, and inappropriate comparison of tools designed for different purposes or target populations. However, there are now several validated, risk scores for all-cause dementia and AD that assess modifiable factors and offer cost-effective dementia risk assessment and risk reduction advice.

## Highlights

• This is the first study explaining heterogeneity in dementia risk score performance.
• Of the 39 dementia risk scores analysed, the pooled AUC is about 0.7.
• Few comparisons between risk scores used valid criteria or consistent exposure age.
• AUCs are higher for development studies than validation studies, creating bias.
• DemNCD, ANU-ADRI, CogDrisk, and LIBRA include most WHO-recommended risk factors.

## 1 Introduction

Given the large projected increase in people with dementia due to population aging [[1], [2], [3]], there is an urgent need to reduce risk and delay or where possible, prevent cognitive decline. Current monoclonal antibody treatments for Alzheimer’s disease (AD) hold promise [4], but they are unlikely to be a panacea for preventing dementia in the short term. This is because most late-life dementia has multiple causes, and the drugs have modest effects, are expensive, and are accessible to a very small minority of patients [4]. Even in a future world where there are effective treatments for the diseases that cause dementia, it will be desirable to address modifiable risk factors that increase the risk of dementia, to optimise brain health, potentially prevent incident cases, and slow disease progression. Similar examples are currently available in the treatment of heart disease, where specialised treatments are available alongside population-level risk reduction strategies [5]. Therefore, addressing modifiable risk factors for dementia needs to be a key long-term and ideally, global strategy for promotion of brain health and prevention of dementia [6]. However, in order to provide risk reduction advice, it is necessary to have accurate and informative risk assessments [7].

There is also now robust epidemiological evidence underpinning a large number of modifiable risk factors for dementia [6,8,9], and randomised controlled trial evidence of the benefits of risk modification for reducing cognitive decline [10], including clinical trial evidence that antihypertensive use is associated with reduced risk of dementia [11]. There has been a recent proliferation of models that combine risk factors to predict AD and dementia [12,13]. Some of these models have been developed into practical tools that are available in digital or other formats for clinicians and the general public [[14], [15], [16]], whilst the majority have remained as risk scores based on published statistical models but not translated for practical day-to-day use. While previous systematic reviews of dementia risk scores have reported on their discriminative performance only through narrative summaries [12,13], key sources of variation, such as, the age at risk assessment, adherence to established guidelines, and the intended target population (e.g., clinical vs. community-based settings), have not been systematically explored. Moreover, prior reviews have not conducted a meta-analysis of discriminative properties of tools, or examined whether variations in the included risk factors contribute to differences in predictive accuracy across tools.

### 1.1 Establishing principles for tool comparison

Previous studies have attempted to compare dementia risk tools, but often these comparisons fail to consider the specific purpose for which tools were designed or the population for which they were developed [12,13,17,18]. Furthermore, evaluations of risk tools have often been approximations of those tools in datasets that do not contain well-defined risk factors, and sometimes they have not been informed by dementia epidemiology. As a result, comparisons are not meaningful or valid. For example, tools developed for midlife may be compared with tools designed for late-life [17]; tools intended for clinical settings, which involve blood biomarkers and imaging, may be compared with tools designed for public use online [18]; or tools may be evaluated on samples with an upper age limit lower than the age at which dementia is most prevalent [19]. The evaluation of risk tools that involve poor measurement of individual risk factors or that are not informed by dementia epidemiology can lead to ‘strawman’ comparisons, and potentially inaccurate results and misleading interpretations.

### 1.2 Need for a framework for describing and evaluating risk assessment tools for dementia

To improve the quality of research in this field, there is a need for an accurate characterisation of the purpose of the dementia risk assessment tool, its target population (age, disease, cohort, location) and the environment in which it is designed for use. This will facilitate a valid comparison of tools that share similar characteristics and purpose. We conducted a systematic review of published risk tools for dementia and summarised their key characteristics to develop findings that would guide the application of risk assessment and guide future research.

The aim of this review is to provide an overview of the published dementia risk assessment tools along with their characteristics and evaluate them according to cost, ease of administration, the degree to which they assess risk factors identified by the World Health Organisation guidelines [10], and to make recommendations for evaluating the utility of dementia risk assessment tools.

## 2 Methods

The systematic review aimed to locate all relevant dementia risk assessment tools. An a priori protocol defining eligibility criteria, search strategy and outcome of interest was developed and was registered with the International Prospective Register of Systematic Reviews (PROSPERO) (CRD42,023,392,435) and reported in accordance with the PRISMA checklist. The population, intervention, comparison, and outcome (PICO) framework to define eligibility criteria was given in the Supplementary Material: PICO Framework section.

### 2.1 Search strategy

Databases PubMed and Cochrane Collaboration, ProQuest, Scopus, Embase, and PsycINFO were searched from inception to 19 February 2025. Reference lists of all papers identified were screened for other published papers. The following terms were used for PubMed: “risk tool*" OR "risk score*" OR "risk assessment*" OR "risk index" OR "risk indices" OR "risk model*”, “valid*" OR "develop*" OR "predict*” "dementia" OR "alzheimer's disease" OR "alzheimer disease", #1 AND #2 AND #3. Searches used for the other databases are reported in the Supplementary materials. The search was limited to articles published in English and in humans.

### 2.2 Selection criteria

The study inclusion criteria for this review were designed to ensure that all articles met the Oxford Centre for Evidence-Based Medicine Level of Evidence 1B (https://www.cebm.net/ = 1025). Only articles that identified a specific dementia risk assessment tool (whether a risk score or another type) were included. These tools had to assess at least some modifiable behavioural factors. Hybrid tools assessing both behavioural and genetic factors were included, but purely genetic risk tools, such as polygenic risk scores, were excluded. Risk scores intended for specific clinical subgroups were allowed. The risk scores had to evaluate all-cause dementia (referred to as "dementia" throughout this review), Alzheimer's disease (AD), or vascular dementia (VaD) as an outcome. The article also had to report information on the validation of the tool and be the first instance of the validation of the tool in the specific type of population. A study could be included if it was validated on a new population, despite having prior validity data published on a different population. Both internal and external validation data were acceptable. Articles had to report a measure of predictive accuracy, such as the area under a receiver operating curve (ROC) curve in relation to dementia incidence, a c-statistic, or risk ratios.

### 2.3 Abstract screening and article selection

Citations downloaded into an EndNote reference database were screened in three stages (title, abstract and full text). The selected journal articles retrieved were examined by at least two of the authors (ML, RE, HH) and rated against the inclusion criteria. Abstracts and articles were discussed with the team (ML, RE, HH, KA) and reassessed where reviewers differed until a consensus was reached. Reference lists of included articles were also searched to identify additional studies for inclusion.

### 2.4 Data extraction

We distinguished between studies reporting the development of dementia risk assessment tools and studies validating risk assessment tools. For each tool, we sought to identify whether it had been validated, externally or internally, either in the original publication or in subsequent publications. External validation could comprise evaluation on a second independent sample, and internal validation could include, for example, a split sample with one part used to develop the tool and one for validation. For each validation study, we extracted data on assessed tools, cohorts used, settings, sample size, minimum and maximum age of the sample assessed, area under the curve (AUC) with 95 % confidence interval (CI), receiver operating characteristics (ROC), if available and the risks factors used in the assessment of the tool.

### 2.5 Classification of tools

To enable classification of tools we coded each one according to the age of assessment (mid-life: ages between 40 and 65, late-life: ages over 65, and mid to late-life: ages spanning between midlife and late-life), target assessment population (e.g. clinical population, general population), and outcome measure (dementia, AD, VaD and cognitive impairment).

In addition, we coded the purpose of the tool, estimated the cost of implementation for each tool and rated the scalability. Costs for administration were based on equivalent costs for services (blood tests, MRI scans, etc.) in Australia. We also coded the risk factors included in each tool to allow for its classification in terms of content, implementation cost, and scalability as well as the extent to which they reflect WHO guidelines related to dementia risk reduction. Risk factors were also coded according to whether they were exogenous of dementia, or whether they could be classified as a ‘risk indicator’. A risk indicator is a variable that indicates cognitive decline or neurodegeneration and hence is not independent of the outcome [20]. Examples of risk indicators include memory tests and neuroimaging indices. Finally, we conducted an online search for each risk tool to locate evidence of implementation of the risk tools via apps or other media.

### 2.6 Statistical analysis

Pooled estimates of AUC for various risk scores assessing dementia, AD and VAD were obtained using a random-effect meta-analysis technique. For specific tools with sufficient data, we also calculated tool-specific pooled AUC values using the same method. The study-specific estimated AUCs were visualized through forest plots. Heterogeneity was examined by using the inconsistency index (I^2^) statistic. Subgroup analyses were conducted to explore sources of heterogeneity by calculating Cochran’s Q statistic. Publication bias was assessed visually using a funnel plot (log AUC against the standard error) using Stata version 16.1. Additionally, we performed a random effects meta-regression to quantify study design characteristics, especially the risk/protective factors, length of follow-ups, and influence on the AUC using R “metaphor” package (R version 4.4.0), adjusting for study and cohort specific random intercepts as well as the mean age of the study participants. When comparing risk scores, we assess the compatibility in terms of age, outcome, and sampling frame.

### 2.7 Quality evaluation

Studies were rated according to the Prediction model Risk Of Bias Assessment Tool (PROBAST) [21] by two independent researchers. PROBAST evaluates the risk of bias and applicability of primary studies that have developed and/or validated diagnostic or prognostic prediction models. Where consensus was not reached between the two assessors regarding PROBAST outcomes, a third reviewer acted as arbitrator.

## 3 Results

### 3.1 Included studies

**Fig. 1:**
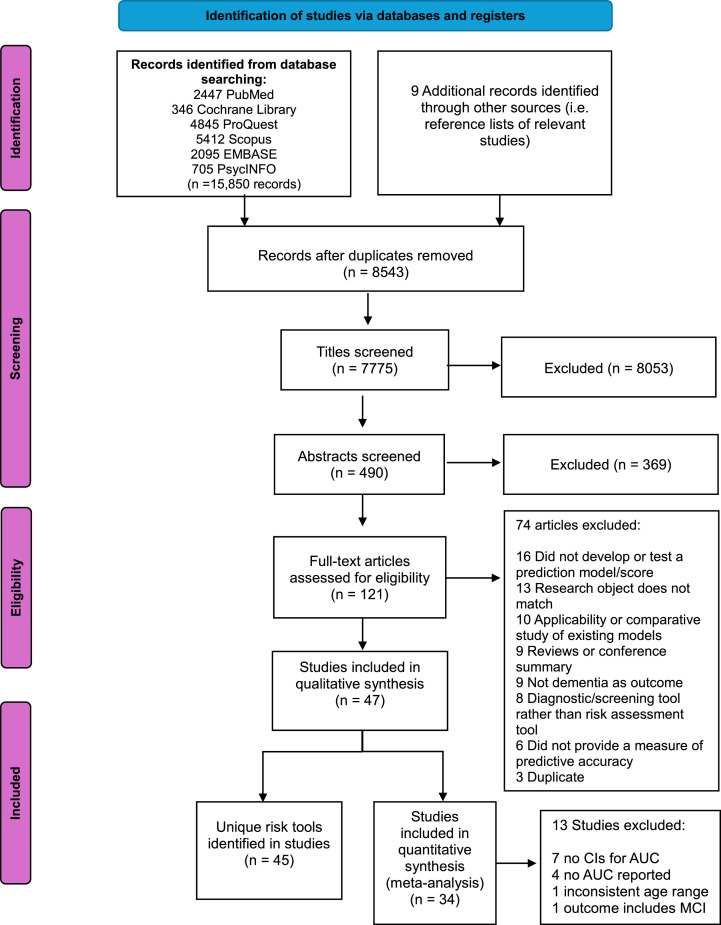
Flowchart for the selection of studies in the systematic review.

Fig. 1 illustrates the process of identifying articles with risk prediction tools and models. In total, 15,850 records were initially identified through searches from database and reference lists. After removing duplicates and title screening, 490 abstracts were screened for eligibility. Among them, 121 full-text articles were screened for eligibility. This resulted in 49 eligible studies with 45 risk scores that met study criteria for inclusion in the review. This includes studies originating from a diverse range of countries, including Australia, New Zealand, the United States, Canada, Finland, Germany, the United Kingdom, Sweden, Spain, Netherlands, Japan, Thailand, Singapore, and Taiwan.

### 3.2 Characteristics of dementia risk tools, their development and validation

#### 3.2.1 Dementia risk scores according to age of assessment

**Fig. 2:**
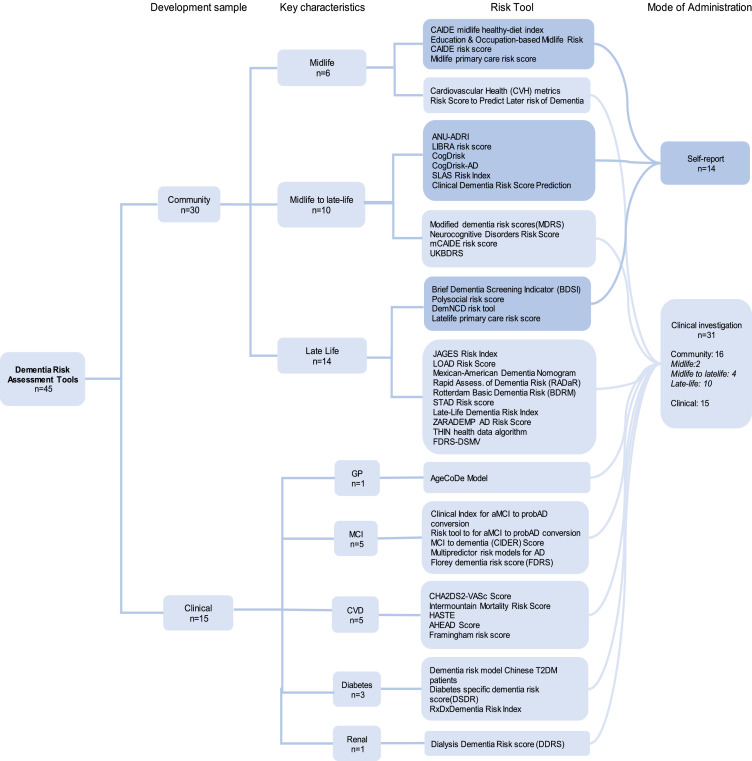
Classification tree for risk tools according to age of risk exposure for development of the tool, setting, and type of administration.

Fig. 2 presents a classification tree for all-cause dementia risk tools based on target population and key sample characteristics. This includes age of assessment (mid-life: ages between 40 and 65, late-life: ages over 65, and mid to late-life: ages spanning between midlife and late-life) for risk tools intended for community use and various predominant clinical characteristics in the development sample for clinical risk tools. Among the 45 risk scores identified in the current study, 6 risk scores were developed using midlife, 10 for midlife to late-life range, 14 for late-life, and 15 were based on clinical risk factor information. All midlife and midlife-late life studies and 48 % (14/29) of the late-life studies were sampled from a community-dwelling population. A majority of the risk scores 67 % (30/45) require clinical investigations to determine the risk scores. Four midlife risk scores: (i) CAIDE risk score [22], (ii) CAIDE midlife healthy diet index [23], (iii) Education and occupation-based midlife risk [24], and midlife primary care risk score [25] can be administered using self-reported risk information. In the mid-to-late life category, six risk scores (Australian National University Alzheimer's Disease Risk Index (ANU-ADRI) [14]; Cognitive Health and Dementia Risk Index (CogDrisk) [15]; CogDrisk for Alzheimer disease (CogDrisk-AD) [15]; LIfestyle for BRAin health (LIBRA) [26]; clinical dementia risk score prediction tool [27]; and SLAS risk index [28]), and in the late-life category, one late-life risk score (BDSI) [29] can be administered using self-reported risk information (see Fig. 2).

#### 3.2.2 Risk scores according to guidelines

Table 1 reports the number of risk factors included in each of the risk scores according to the WHO guidelines for dementia [10]. Among the 12 risk factors identified in the guideline, the DemNCD incorporated the highest number of WHO identified risk factors (*n* = 11), followed by ANU-ADRI, LIBRA, CogDrisk and CogDrisk-AD, each including 10 risk factors. The ‘Risk scores for late-life dementia’ [30], both midlife and late-life dementia risk models in primary care [31] and the ‘THIN health data algorithm’ [32] each included seven risk factors identified in the WHO guideline. Five risk scores included four, three included three, two included only one risk factor, and seven of the risk scores included none of the WHO risk factors in the risk score development.

**Table 1:** The number of risk factors included in risk scores for which recommendations were made in the WHO guidelines.

#### 3.2.3 Risk score according to the underlying population characteristics and outcome of interest

Out of 45 risk scores, 29 risk scores were developed to predict the risk of all-cause dementia, 10 for AD, 5 for cardiovascular disease (CVD) and 1 for cognitive impairment (combining MCI and dementia) (see Supplementary Table 1). Of the 29 dementia risk scores, 24 were derived using community samples, including 11 based on late-life cohorts, 6 from midlife, and 7 utilizing a range of risk factors spanning from midlife to late-life. The remaining 5 risk scores were developed using clinical samples (Supplementary Table S1). The mean age of participants in the development sample for midlife risk scores ranges from 46 to 57 years, with follow-up durations varying from 21.5 years to 30 years. In contrast, the mean age ranges from midlife to late-life, late-life community sample and clinical samples are 56.3–67.9, 71.2–79.7 and 65.2–77.7, respectively. The mean follow-up durations for these groups are 0–14 years, 2–20 years and 0.5–9 years, respectively. One late-life risk tool, the mCAIDE [33] was developed using a cross-sectional study design to predict cognitive impairment. In addition, 4 midlife to late-life risk tools (ANU-ADRI, LIBRA, CogDrisk and CogDrisk-AD) were developed through evidence synthesis.

#### 3.2.4 Risk factor distribution in risk scores

**Fig. 3:**
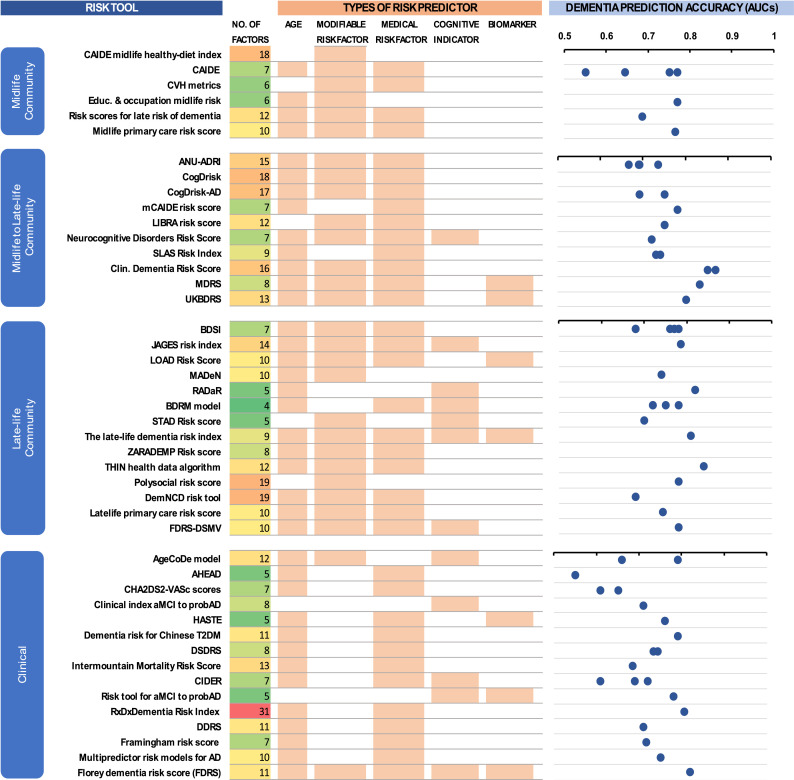
Gopher chart of risk scores and their general composition.

The number of risk factors included in each risk score varied, ranging from 5 to 31 risk factors in each tool, with a median of 8 risk factors (Fig. 3). Table 2 presents the distribution of risk and protective factors included in the development of these risk scores. Most of the risk scores included participants’ age (84 %) followed by sex (64 %), diabetes (62 %), education (51 %), hypertension (53 %), depression (44 %), BMI/Obesity (40 %), dyslipidaemias (36 %), physical inactivity (36 %), measures of cognitive function (34 %), and smoking (33 %). Information on diet and APOE ε4 were included in 8 risk scores, while 4 risk scores required data from MRI and cognitive activity, and 3 risk scores needed information from pesticide exposures. Age was included in almost all AD risk scores (9 out of 10), whereas 23 out of 29 dementia risk scores included age. Notably, 10 dementia risk scores included cognitive function in the development, i.e., as part of the risk calculation, although cognitive function is an indicator of dementia rather than a risk factor.

**Table 2:** Distribution of risk and protective factors included in the development of these risk scores.

		Outcome measures			Age characteristics			
Variables	All risk scores^†^	Dementia	AD	CVD	Midlife	Midlife to late-life	Late-life	Clinical
	*n* = 45 ( %)	*n* = 29 ( %)	*n* = 10 ( %)	*n* = 5 ( %)	*n* = 6 ( %)	*n* = 10 ( %)	*n* = 14 ( %)	*n* = 15 ( %)
Age	38 (84.4)	23 (79.3)	9 (90.0)	5 (100.0)	3 (50.0)	9 (90.0)	12 (85.7)	14 (93.3)
Sex	29 (64.4)	18 (62.1)	7 (70.0)	3 (60.0)	3 (50.0)	9 (90.0)	9 (64.3)	8 (53.3)
Education	23 (51.1)	14 (48.3)	6 (60.0)	2 (40.0)	2 (33.3)	8 (80.0)	7 (50.0)	6 (40.0)
Diabetes/Glucose	25 (55.6)	18 (62.1)	4 (40.0)	3 (60.0)	4 (66.7)	7 (70.0)	8 (57.1)	7 (46.7)
BMI/Obesity	18 (40.0)	14 (48.3)	3 (30.0)	1 (20.0)	4 (66.7)	6 (60.0)	6 (42.9)	2 (13.3)
Hypertension	24 (53.3)	16 (55.2)	5 (50.0)	3 (60.0)	4 (66.7)	6 (60.0)	3 (21.4)	8 (53.3)
Cholesterol/HDL/LDL	16 (35.6)	12 (41.4)	3 (30.0)	1 (20.0)	4 (66.7)	6 (60.0)	3 (21.4)	3 (20.0)
Hearing loss	4 (8.9)	2 (6.9)	1 (10.0)	0 (0.0)	0 (0.0)	1 (10.0)	3 (21.4)	0 (0.0)
Sleep problem	3 (6.7)	3 (10.3)	0 (0.0)	0 (0.0)	0 (0.0)	1 (10.0)	1 (7.1)	1 (6.7)
Traumatic Brain Injury (TBI)	5 (11.1)	3 (10.3)	2 (20.0)	0 (0.0)	0 (0.0)	3 (30.0)	1 (7.1)	1 (6.7)
Depression	20 (44.4)	14 (48.3)	5 (50.0)	0 (0.0)	0 (0.0)	7 (70.0)	9 (64.3)	4 (26.7)
Other medical conditions^‡^	18 (40.0)	12 (41.4)	3 (30.0)	3 (60.0)	0 (0.0)	5 (50.0)	6 (42.7)	6 (40.0)
Physical activity	16 (35.6)	11 (37.9)	4 (40.0)	1 (20.0)	3 (50.0)	6 (60.0)	5 (35.7)	2 (13.3)
Cognitive function	15 (33.5)	10 (34.5)	5 (50.0)	0 (0.0)	1 (16.7)	1 (10.0)	7 (50.0)	6 (40.0)
Smoking	15 (33.3)	9 (31.0)	6 (60.0)	0 (0.0)	2 (33.3)	6 (60.0)	5 (35.7)	2 (13.3)
Diet	8 (17.8)	5 (17.2)	3 (30.0)	0 (0.0)	2 (33.3)	4 (40.0)	2 (14.3)	0 (0.0)
Social engagement	9 (20.0)	5 (17.2)	4 (40.0)	0 (0.0)	0 (0.0)	3 (30.0)	5 (35.7)	1 (6.7)
Alcohol	8 (17.8)	7 (24.1)	1 (10.0)	0 (0.0)	2 (33.3)	2 (20.0)	4 (28.6)	0 (0.0)
Cognitive engagement	4 (8.9)	2 (6.9)	2 (20.0)	0 (0.0)	0 (0.0)	3 (30.0)	1 (7.1)	0 (0.0)
Apoe ε4	8 (17.8)	4 (13.8)	4 (40.0)	0 (0.0)	1 (16.7)	1 (10.0)	3 (21.4)	3 (20.0)
MRI	4 (8.9)	2 (6.9)	1 (10.0)	1 (20.0)	1 (16.7)	0 (0.0)	1 (7.1)	2 (13.3)
Pesticides	3 (6.7)	0 (0.0)	3 (30.0)	0 (0.0)	0 (0.0)	2 (20.0)	1 (7.1)	0 (0.0)

Note: *includes 29 scores for dementia, 10 for AD, 5 for CVD, and 1 for MCI/dementia.

^‡^Other medical condition includes wide range of medical condition such as stroke, Atrial Fibrillation, Coronary heart disease, Myocardial Infraction, Arthritis, cerebrovascular disease, cardiovascular disease, history of bypass surgery, Chronic Kidney disease, renal failure, Parkinson’s, cancer etc.

#### 3.2.5 Characteristics of the validation of risk scores

Supplementary Table 2 presents detailed characteristics of validation studies for various dementia risk scores. Sixteen risk prediction models (including 10 for dementia, 5 for AD, and 1 for CVD) were not validated using any internal or external validation samples. The remaining 29 risk prediction models were validated using 35 studies (with a range of 1 to 5 studies per model). Among the validated risk scores, CAIDE was validated on the highest number of external studies (validated in 5 studies), followed by LIBRA and ANU-ADRI, each validated in 4 studies. The CogDrisk, CogDrisk-AD, and the Framingham CVD risk scores were each validated in two studies. All other risk scores were validated in a single either external or internal study (Supplementary Table S2). The midlife risk prediction tool CAIDE was validated using data from midlife (2 studies), late-life (2 studies) and clinical samples (1 study). Similarly, the mid-to-late-life risk tool, LIBRA was validated using midlife (2 studies), mid-to-late-life (1 study) and late-life samples (3 studies). Although the ANU-ADRI risk tool was developed for risk assessment of AD, in addition to validation for AD (2 studies), it has been validated for dementia risk assessment using both late-life (2 studies) and mid-to-late-life risk information (1 study).

### 3.3 Meta-analysis of the predictive accuracy of the development and validation sample

**Fig. 4:**
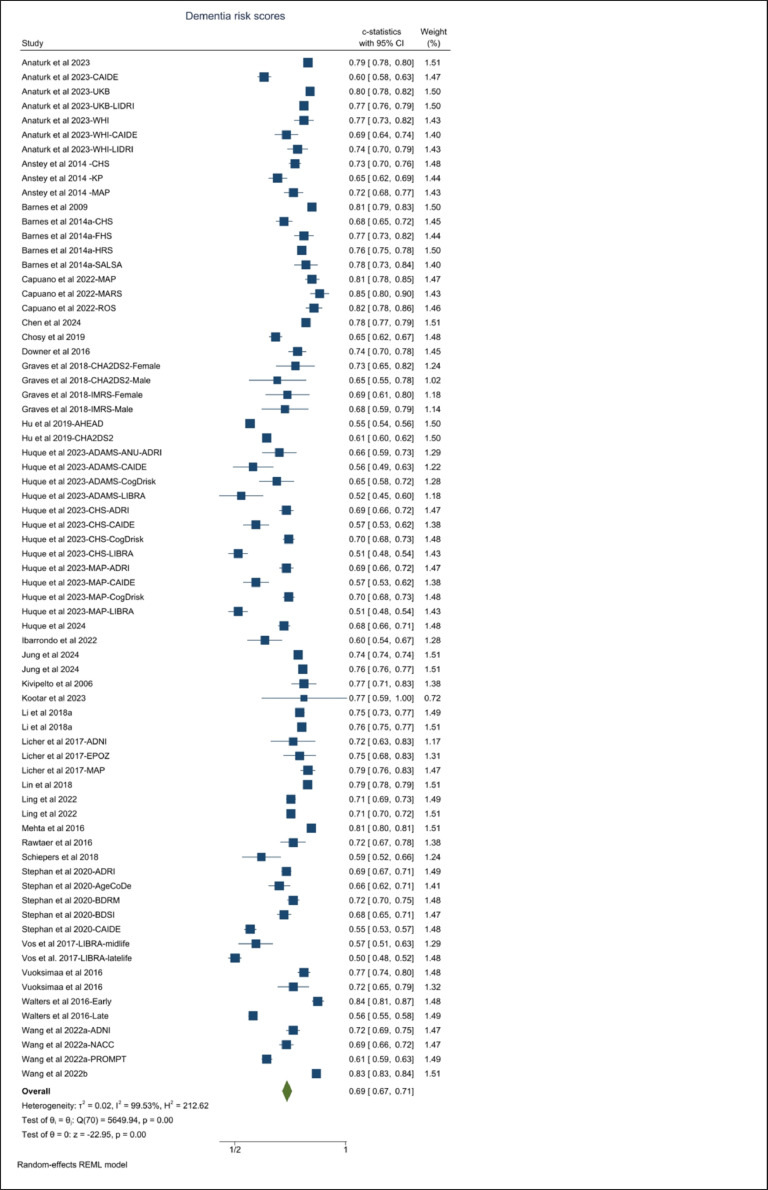
Forest plot of AUCs/C-statistics obtained from meta-analysis using all dementia risk scores.

The mean age and length of follow-up, along with detailed sample characteristics for the development and validation of risk scores, are provided in Supplementary Table S1 and Supplementary Table S2, respectively. Pooled c-statistics (95 % CI) for various risk scores, based on both development and validation samples are presented using forest plots in Fig. 4 and Supplementary Figures S1-S6. The combined c-statistic (95 % CI) of all dementia risk scores in the validation and development sample is 0.69 (0.67, 0.71) (Fig. 4).

#### 3.3.1 Performance of midlife risk scores in predicting dementia risk

The mean age of the development samples for midlife risk prediction scores ranged from 46 to 57 years, with follow-up durations from 8.3 to 31 years. Among the validation studies, three studies that validated midlife risk scores had follow-up intervals of 20 to 39 years [24,30,34], three had shorter follow-up periods of 0, 3.8, and 5 years. Out of the six midlife risk scores, three reported AUC with 95 % CI, yielding a pooled estimate of 0.71 (0.61, 0.83). Among these, the CAIDE, and the Education and occupation-based midlife risk score were validated externally, resulting an estimated c-statistic (95 % CI) of 0.63 (0.58, 0.68) (Supplementary Figure S1). Note that studies using midlife data [19,24,35] to validate midlife risk scores provided better c-statistics than those using late-life data [19,25,36,37].

#### 3.3.2 Performance of midlife to late-life risk scores in predicting dementia risk

For mid-to-late-life risk models, the mean age of the development samples ranges from 56 to 68 years, with follow-up durations from 0 to 14 years. The mean age of validation samples for this category ranged from 57 to 74 years, with follow-up also from 0 to 14 years. Notably, four mid-to-late-life risk tools (ANU-ADRI, LIBRA, CogDrisk and CogDrisk-AD) were developed using evidence synthesis and validated across diverse cohorts, with mean participant ages ranging from 47.85 to 80 years and follow-up durations from 0 to 30 years. Apart from a cross-sectional study and a study with long follow-up (30 years), all other validation studies had follow-up ranges from 5 to 16 years.

Among the 10 risk scores utilizing midlife to late-life risk factors, 6 were developed using cohort data, yielding a pooled c-statistic (95 % CI) of 0.79 (0.73, 0.85) (Supplementary Figure S1). Note that two of the three studies used to estimate the pooled estimate of the development sample were from the UK Biobank. The other UK Biobank study provides similar c-statistics but was not included in the meta-analysis due to the absence of a confidence interval for the c-statistic estimate in the study [27]. Only the UKBDRS risk score that was developed using cohort data was externally validated. The estimated c-statistics for the midlife to late-life tools (developed from cohorts and evidence synthesis) on the validation sample is 0.65 (0.60, 0.69). Although the LIBRA tool uses risk information from both midlife and late-life, it did not include non-modifiable risk factors such as age, sex and education, resulting in low c-statistics in validation. Moreover, studies that use midlife to late-life samples [19,38] for the validation of these risk tools exhibited better c-statistics compared to studies that used late-life samples (Supplementary Figure S1).

#### 3.3.3 Performance of late-life risk scores in predicting dementia risk

For late-life risk scores, the mean age of the development sample ranged from 71 to 80 years, with follow-ups lasting 2 to 20 years. Mean participants’ ages in the validation samples ranged from 57 to 76 years, with follow-up durations from 2 to 18 years. All of the community-dwelling samples used to validate late-life risk tools had follow-ups ranging from 5 to 10 years, except for the UK Biobank [19], Whitehall [19], and ROS cohorts [39]. Note that the UK Biobank includes participants from both midlife and late-life, however, the Whitehall study includes participants from midlife.

Among the 14 late-life risk scores that were developed using late-life community samples, only 7 reported AUC/C-statistics for dementia in the development samples, resulting in pooled estimated c-statistics (95 % CI) 0.77 (0.75, 0.80). The pooled c-statistics (95 % CI) for the validation samples is 0.73 (0.69, 0.79) (Supplementary Figure S2).

#### 3.3.4 Performance of clinical risk scores in predicting dementia risk

For the clinical risk scores, the mean age of the development samples ranged from 65 years to 80 years, with follow-up durations of 0.5 years to 12 years; validation sample ages ranged from 67.2 to 73.9 years, with follow-up durations of 2.91 to 12 years. Only two validation studies had follow-up durations of <3 years.

Among the 15 clinical risk scores, five were developed to estimate the risk prediction for all-cause dementia, 5 for AD, 4 for CVD, and 1 for VAD. Evidence on the performance of CVD risk for dementia risk tools is only available through validation studies. Of the clinical risk scores for dementia, one risk score did not report a confidence interval for AUC. The pooled c-statistics for the clinical risk scores for dementia in development samples were 0.74 (0.69, 0.79) and 0.66 (0.62, 0.71) in validation samples (Supplementary Figure S2). Risk scores that utilized a large number of clinical markers or registry data tended to result in higher AUCs.

#### 3.3.5 Performance of widely validated risk tools of dementia

The performance of risk tools evaluated in multiple studies, such as CAIDE, LIBRA, ANU-ADRI, CogDrisk, and BDRM, is illustrated in Supplementary Figure S3. The pooled estimates for the CAIDE risk score in the validation sample is 0.60 (0.56, 0.64), with better performance observed in studies using midlife risk factors. The pooled c-statistic for LIBRA is 0.52 (0.50, 0.55), likely due to the exclusion of age, sex, and education. Both CogDrisk and ANU-ADRI demonstrated similar predictive performance, with pooled AUCs of 0.70 (0.68, 0.72) and 0.69 (0.68, 0.71), respectively. The Basic Dementia Risk Model (BDRM) [40] yielded a pooled c-statistics of 0.72 (0.70, 0.75), while the Late-Life Dementia Risk Index reported a pooled c-statistics of 0.72 (0.61, 0.85). Note that the BDRM risk tool includes indicators of both subjective memory decline and IADL.

#### 3.3.6 Subgroup analysis according to included risk factors

Only a few studies incorporated APOE ε4, physical activity, cognitive activity, diet and cognitive function. Supplementary Figure S4 demonstrates that risk scores including APOE ε4 and cognitive function resulted in higher c-statistics - 0.74 (0.68, 0.80) for APOE ε4 and 0.73 (0.70, 0.76) for cognitive function - compared to the overall c-statistics 0.69 (0.67, 0.71). In contrast, studies that included physical activity or cognitive activity in risk prediction resulted in wide range AUCs.

#### 3.3.7 Alzheimer’s disease (AD) risk scores

Supplementary Table S3 lists all AD risk factors considered in the development AD risk scores across community and clinical settings. AD risk scores from clinical settings predominantly utilized cognitive function and functional dependence indicators, while community-based AD risk scores incorporated a broader range of risk factors. The pooled c-statistics for the AD risk tools in development samples is 0.79 (0.76, 0.82) and it is 0.71(0.68, 0.75) in validation samples (Supplementary Figure S5). Among these, the ANU-ADRI and CogDrisk-AD have been validated in a number of studies, providing similar estimated pooled c-statistics (supplementary Figure S6). Of all the tools reviewed, these were the only ones to include environmental risk factors, namely exposure to pesticides.

#### 3.3.8 Vascular dementia (VaD) risk scores

Only two studies presented risk scores for vascular dementia (VaD), one with clinical sample [41], and one using UK Biobank dataset [42] with AUCs (95 % CI) 0.76 (0.69, 0.83) and 0.85 (0.84, 0.87). However, none of these scores are validated.

#### 3.3.9 Contribution of various factors in the performance of dementia risk scores

Length of follow-up, sample size, inclusion of certain covariates and prevalence of dementia in the sample all impact the performance of the tool and preclude direct comparison of AUCs between publications, we therefore conducted meta-regression to evaluate how the study design influences the reported AUC in the literature. Table 3 presents results from the meta-regression on the effect of including various risk factors on log (AUC) estimates for both community and clinical sample based risk scores. Our results showed that inclusion of BMI in risk scores for late life significantly increases AUC (average increase 26 %), on the contrary the inclusion of dyslipidaemia significantly decreases AUC (average decrease 26 %) in late life risk scores. Among all risk scores, age in risk scores increases AUCs (average increase 27 %−42 %) for community sample based dementia risk scores. Conversely, inclusion of physical activity in risk scores decreases AUCs (average decrease 12 %) for community sample based dementia risk scores, however, none of these estimates are statistically significant.

**Table 3:** Meta regression analysis of log (AUC) associated with covariates in risk scores.

	Community sample				Clinical
	Dementia			AD	Dementia
	All sample^†^	Midlife to late-life	Late life	All sample^†^	All sample^†^
Variables	$e^{\beta }\left(95\%\;\text{CI}\right)$	$e^{\beta }\left(95\%\;\text{CI}\right)$	$e^{\beta }\left(95\%\;\text{CI}\right)$	$e^{\beta }\left(95\%\;\text{CI}\right)$	$e^{\beta }\left(95\%\;\text{CI}\right)$
Age	1.27 (0.94, 1.72)	1.41 (0.16, 12.31)	1.42 (0.90, 2.23)	1.05 (0.02, 5.86)	0.93 (0.75, 1.16)
Sex	1.06 (0.86, 1.32)	NAN	1.11 (0.68, 1.80)	0.91 (0.60, 1.39)	0.92 (0.67, 1.27)
Education	0.97 (0.82, 1.15)	0.98 (0.09, 11.06)	0.87 (0.71, 1.08)	1.30 (0.17, 9.80)	NAN
APOE ε4	1.10 (0.91, 1.34)	1.22 (0.20, 7.62)	1.03 (0.63, 1.70)	0.99 (0.51, 1.91)	NAN
Hypertension	1.03 (0.85, 1.25)	1.05 (0.60, 1.83)	0.95 (0.71, 1.28)	0.85 (0.14, 5.04)	1.20 (0.83,1.75)
Dyslipidemia	0.92 (0.78, 1.08)	1.03 (0.40, 2.68)	**0.74 (0.58, 0.96)**	0.78 (0.01, 6.11)	1.13 (0.84, 1.51)
Diabetes	1.09 (0.85, 1.39)	1.31 (0.09, 19.60)	1.11 (0.76, 1.63)	0.90 (0.08, 9.81)	0.77 (0.62, 0.95)
BMI/Obesity	1.00 (0.87, 1.15)	0.92 (0.20, 4.28)	**1.26 (1.02, 1.55)**	1.00 (0.29, 3.44)	NAN
Hearing loss	1.01 (0.74, 1.38)	NAN	1.25 (0.80, 1.95)	NAN	NAN
Sleep problem	0.93 (0.67, 1.28)	0.95 (0.33, 2.69)	0.78 (0.46, 1.32)	0.98 (0.21, 4.62)	NAN
Traumatic brain Injury	1.03 (0.76, 1.39)	0.92 (0.42, 2.00)	0.87 (0.59, 1.27)	0.94 (0.20, 4.45)	NAN
Other medical condition^‡^	1.01 (0.87, 1.19)	1.04 (0.80, 1.35)	1.03 (0.72, 1.49)	1.00 (0.54, 184)	NAN
Cognition	1.04 (0.83, 1.30)	NAN	1.02 (0.75, 1.38)	1.17 (0.20, 7.02)	NAN
Social engagement	0.99 (0.82, 1.20)	0.98 (0.47, 2.06)	1.06 (0.82, 1.37)	1.00 (0.45, 2.24)	NAN
Cognitive engagement	1.06 (0.75, 1.48)	1.02 (0.29, 362)	1.23 (0.75, 2.03)	1.11 (0.62, 1.99)	NAN
Depression	0.93 (0.74, 1.17)	0.97 (0.22, 4.36)	0.81 (0.59, 1.11)	0.89 (0.20, 3.90)	NAN
Alcohol	0.95 (0.75, 1.20)	0.96 (0.59, 1.58)	0.84 (0.54, 1.30)	0.98 (0.39, 2.48)	NAN
Smoking status	1.04 (0.87, 1.22)	1.00 (0.03, 29.31)	1.19 (0.92, 1.54)	1.08 (0.22, 5.25)	NAN
Physical activity	0.88 (0.74, 1.04)	1.08 (0.40, 2.89)	0.94 (0.72, 1.21)	NAN	NAN
Diet	1.12 (0.87, 1.45)	1.07 (0.40, 2.84)	1.21 (0.79, 1.86)	NAN	NAN
Follow-up duration				NAN	
0–5 years	Ref	Ref	Ref		Ref
6–10 years	1.07 (0.89, 1.22)	0.89 (0.15, 5.37)	1.08 (0.86, 1.35)		NAN
>10 years	1.03 (0.86, 1.22)	NAN	1.13 (0.91, 1.40)		NAN
Cohorts type		Not included	Not included	NAN	NAN
Midlife	Ref				
Midlife to late-life	1.00 (0.86, 1.18)				
Late-life	0.88 (0.70, 1.11)				

Note: *adjusted for mean age.gal.

†NAN: Not included in the final model as redundant.

^‡^Other medical condition includes wide range of medical condition such as stroke, Atrial Fibrillation, Coronary heart disease, Myocardial Infraction, Arthritis, cerebrovascular disease, cardiovascular disease, history of bypass surgery, Chronic Kidney disease, renal failure, Parkinson’s, cancer etc.

### 3.4 Risk of bias assessment in dementia risk prediction models

The risk of bias assessment using the Prediction model Risk of Bias Assessment Tool (PROBAST) is provided in Supplementary Table S4. The PROBAST tool evaluates studies across four domains for risk of bias and three domains for the applicability of the risk prediction model.

Among the 49 studies included in this review, 41 (84 %) were classified as having a high risk of bias, five (10 %) as low risk, and three (6 %) as having an unclear risk of bias. Notably, biases related to participants, predictors, or outcomes were observed in only a subset of studies, whereas most of the high-risk classifications were due to issues in the analysis domain. Common sources of bias in analysis included: excluding participants from the final analysis; selecting predictors based on bivariate analysis with arbitrary p-value thresholds; improper handling of missing data or censoring; and failure to account for overfitting in model development.

Of the five studies [30,32,36,38,40] assessed as having a low risk of bias, two involved both development and validation of risk scores, two were external validations of existing tools, and one focused solely on tool development. One study, which developed the RXDx Dementia Risk Score [43], provided insufficient information on the ascertainment of outcomes and predictors, and thus the risk of bias could not be assessed. Additionally, CogDrisk, CogDrisk-AD [15], and ANU-ADRI [14] were developed through evidence synthesis rather than primary cohort data. As a result, PROBAST’s cohort-specific bias domains were not applicable to these tools, leading to their classification as having unclear risk of bias.

### 3.5 Administration of dementia risk tools

#### 3.5.1 Information required for implementation

Three midlife assessment tools could be administered by self-report [[22], [23], [24]], and the other midlife risk tools [30,44] required clinical tests such as clinical assessments, blood tests, and/or genotyping. Note that the CAIDE risk tool has two versions, one can be administered by self-report and the other requires genotyping. Of risk scores developed on midlife to late-life samples, seven could be administered purely by self-report [14,15,19,[26], [27], [28],^45^], whereas five required clinical assessments and/or additional blood tests or imaging. Of the late-life risk tools, three could be administered using self-reported data [29,32,[46], [47], [48], [49]]. Of the tools developed on clinical samples, all involved either clinical tests or administrative data. There were two derived from administrative databases [50,51], two required brain imaging [41,52]and several required blood tests.

#### 3.5.2 Economic cost and potential scalability

**Fig. 5:**
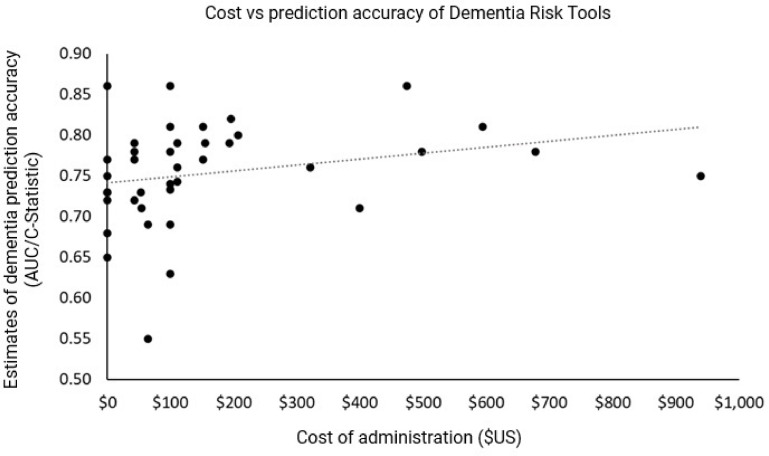
Cost of tools plotted against AUC statistic. Note this does not take into account length of follow-up or the age distribution of samples, which may influence AUCs.

The costs of dementia risk assessment varied from zero to approximately $939USD (Fig. 5), noting that costs do not take into account the time of the participant (see supplementary Table S5) and are based on the Australian Medical Benefits Scheme rebates. These results give a broad indication of the relative cost of tools. Those involving MRIs and memory clinics were the most expensive. Fig. 3 shows the AUCs plotted against the cost of risk assessment tool. There appears to be no association between cost and predictive accuracy, although this figure does not take into account other factors such as length of follow-up or age.

## 4 Discussion

This systematic review provides a comprehensive overview of 45 published dementia risk scores and critically evaluates their content, validation, cost, adherence to WHO guidelines, and generalisability. The meta-analysis revealed that the pooled estimate for the AUC of dementia risk scores is around 0.7. A new risk tool for dementia developed from 10 cohorts studies also provided similar pooled AUCs [45]. Moreover, we did not find greater predictive validity when including biomarkers, which may be expensive, in risk models that also included preventative risk factors. However, a recent study focusing only on biomarkers suggests higher AUCs for plasma biomarkers in predicting dementia [53], which needs further verification in independent studies. Plasma biomarkers of disease are inherently different from modifiable risk factors which are independent of disease processes, and risk scores comprised of plasma biomarkers may complement prevention focussed risk assessment.

Our review identified a handful of dementia risk scores considered modifiable risk factors for dementia that are considered in the WHO guidelines. Two of the five midlife risk tools included information on age, sex, education, physical activity, diet, and smoking. A small number of late-life risk tools included modifiable risk factors such as physical inactivity, cognitive engagement, social engagement, and diet. Similarly, many midlife-to-late-life risk scores did not include information on diet, social engagement, or cognitive engagement. As a result, their effectiveness as risk monitoring tools for targeting strategies for delaying cognitive decline and dementia in late life may be questionable, as optimal social and cognitive engagement may help delay the progression of dementia. Risk scores that include modifiable risk factors [36], [45], are not only effective and inexpensive but also aligned with the factors identified by the WHO guidelines [6].

There was also overlap with the risk factors identified in the Lancet Commission on Dementia [6]. Interestingly, 15 risk scores included high cholesterol, reflecting the evidence for this factor in the literature over a long period of time [54], despite it only being included in the most recent Lancet Commission list of key risk factors. Three studies included hearing loss despite the growing evidence of its role as a risk factor (although there is limited evidence at this stage of the benefits of intervention). No study included uncorrected vision loss [6]. Despite the importance of low education as a risk factor, only 50 % of late life risk tools, and 40 % of clinical tools included this but 80 % of midlife risk tools included education. This may reflect the emphasis on modifiable risk factors in the tools reviewed. While the Lancet Commission did not include an unhealthy diet in its of risk factors, the WHO did make recommendations for healthy diet reducing risk of cognitive decline and dementia and diet was included in 40 % of midlife risk tools, and 14.3 % of late-life risk tools. Overall these differences show that tools that measure modifiable risk factors will need to be updated as the evidence base continues to grow and clarify.

Our results highlight that self-reported risk assessment tools (e.g., CAIDE, LIBRA, BDRM, CogDrisk, ANU-ADRI) are widely validated tools across diverse settings and exhibit good performance in the validation samples (see Supplementary Figure S3) [35,36,55,56]. The CogDrisk ANU-ADRI, and LIBRA were developed using evidence synthesis can only be evaluated through validation samples. Hence, these tools are often misrepresented when compared with others that are not fully aligned with their development principles or with dementia epidemiology. For example, the LIBRA score does not include effects of non-modifiable risk factors such as age, sex and education, and ANU-ADRI assumes a weight of zero for ages under 65. Hence, comparing these risk tools with other risk tools, which provide risk stratification based on age in midlife results in an invalid comparison. The CAIDE risk tool has been developed using age weights of midlife samples (age<65). It is well known that age is a significant risk factor for dementia, and prevalence as well as incidence of dementia increase with increasing age [57]. Therefore, comparing CAIDE risk scores with other risk scores that were developed using late-life age weights can lead to misleading conclusions. All of the risk scores included in our review are specific to either midlife or late life. More recently, a midlife version of the CogDrisk tool, CogDrisk-ML [58], was developed, which can be used in combination with the original CogDrisk tool for late life. Together, these tools enable dementia risk prediction across the life course, from midlife to late life.

The age at which risk factors are assessed is critical, prevalence of dementia increases with age [57], and the relevance of risk factors can change over the life course [59]. For example, hypertension, high BMI and serum cholesterol may not increase risk of dementia when they are observed in late-life [8]. Moreover, the salience of risk factors in the oldest old (adults aged over 85 years) is not well understood. Additionally, if follow-up periods do not extend into very old age, the true incidence of dementia in the population will not be established, as seen in the UK Biobank, where high AUCs can be attributed to age alone rather than the full model [19,27,42,[60], [61], [62]]. This is because the UK Biobank study includes samples from age 44 to 73, with a 14-year follow-up; hence, individuals in the upper age spectrum of this cohort experience dementia at a higher rate than participants in the lower age spectrum. As a result, in the UK Biobank, age alone has a higher predictive power for dementia than in other cohorts, which results in relatively higher AUCs. For example, previous report suggests that CogDrisk-AD and ANU-ADRI provide similar but moderate AUCs (AUC ranges from 0.65 to 0.72) for the prediction of AD when applied to the Rush Memory and Aging Project (MAP), the Cardiovascular Health Study Cognition Study (CHS-CS), and the Health and Retirement Study–Aging, Demographics and Memory Study (HRS-ADAMS) [36,55]. However, application of CogDrisk-AD on UK-Biobank data resulted an AUC of 0.856 [63]. Moreover, the recent Lancet commission report [6] estimated that up to 45 % of dementia cases are attributable to 14 modifiable risk factors. None of the risk scores reviewed here included all the risk factors identified in the Lancet Commission. Altogether, this raises the concern that AUCs for dementia risk scores exceeding 0.8 may be an artifact of exceptionally wide age ranges in cohorts or sample bias.

In addition, we found that the inclusion of physical activity as a risk factor was associated with lower AUCs in community-based samples assessing dementia risk. Among the studies that included physical activity, only 7 % also incorporated a measure of cognitive function. In contrast, approximately 50 % of studies included cognitive assessments without considering physical activity. Since cognitive assessments are closely linked to dementia outcomes and often serve as direct indicators of the condition, these studies tended to report higher AUCs. As a result, the observed differences in model performance may be biased due to confounding by cognitive measures. Similarly, late-life studies that included dyslipidaemia as a risk factor were less likely to include cognitive assessments in their risk scores, which may have contributed to the lower AUCs observed in these studies compared to those that did not include dyslipidaemia. Notably, a slightly higher proportion of risk scores that included BMI or obesity measures also incorporated cognitive assessments and other medical conditions such as stroke, Atrial Fibrillation, heart disease, renal failure etc.. This highlights the importance of critically evaluating the components of risk scores when comparing their predictive performance.

Our review had both strengths and limitations. To our knowledge, this is the most comprehensive review of dementia risk scores in terms of the domains that were evaluated. The review identified very few studies comparing different risk tools using valid criteria and consistent age of exposure. A strength is the finding of overall consistency in the effect sizes for dementia risk assessment tools identified throughout the review. We identified several methodological limitations in validation studies, including inappropriate age or exposure, age ranges and length of follow-up, and small sample sizes. The use of clinical samples introduces selection bias, which precludes generalisation to the population, unless a tool is externally validated on a population sample. Another observation was that inappropriate comparisons made in validation studies can lead to inaccurate or misleading conclusions (e.g. evaluating a tool developed for late-life, on the UK Biobank/Whitehall sample, which has a midlife sample) [19]. In our review, we included a relatively smaller number of studies compared to previous reviews [12,13], reflecting our distinct objectives. Whereas prior reviews incorporated a larger number of studies using artificial intelligence (AI), machine learning (ML) algorithms, or clinical risk assessments, we focused specifically on models with greater potential for real-world implementation in guiding dementia risk reduction strategies. Many AI/ML-based models lack external validation, have limited transportability, and do not consistently outperform traditional statistical approaches [12]. Moreover, they often fail to offer actionable insights relevant to prevention, which is the central focus of our review. Similar to previous reviews [12,13], we also found that a large number of risk scores were not validated using any internal or external datasets. Therefore, concerns remain regarding the heavy dependence on a single data source and the lack of sufficient validation in these models. Similar findings were also reported elsewhere [52]. Our risk of bias assessment reveals that only handful of risk scores are of low risk of bias which indicate severe methodological flaws, and need greater care for future development and validation of dementia risk assessment tools.

In our review, we observed considerable variation in the number of risk factors included across different dementia risk tools. Therefore it might be interesting to explore whether including a greater number of risk factors, particularly those identified by the WHO or Lancet Commision leads to improved predictive performance. Addressing this question would require cohort datasets that include comprehensive information on all relevant risk factors and maintain consistent age distributions. We suggest that future studies explore this issue in greater detail.

A key aspect of dementia risk tools that requires better definition is the purpose of the tool. Some risk tools were developed as statistical models without a clear plan for use in clinical practice. Others were developed for use in clinics and others for primary prevention and primary care. If the goal is to maximise statistical prediction, then the specific risk factors included (e.g. risk indicators) do not matter. However, if the tool has been developed with the aim of providing information for practical risk reduction, then it is essential that the tools include relevant factors that can effectively guide intervention.

## 5 Conclusion

Dementia is a major cause of disability and dependence in older adults, highlighting the urgent need to identify those at high risk early. Numerous dementia risk scores have been developed as statistical models, with a few being adapted into affordable risk assessment tools. Risk scores that include modifiable risk factors offer effective, inexpensive and valid risk assessment and align with dementia risk reduction guidelines, making them a practical approach for prevention.

## Source of funding

This project is funded by NHMRC GNT1171279. KA is funded by ARC laureate Fellowship FL190100011.

## Author’s contribution

KA developed the project, wrote the first draft and supervised the project. HH, RE and ML conducted systematic review of literature. HH and ML independently extracted the data. HH conducted the statistical analysis and drafted sections of the results. HH, RE, ML, and KA prepared figures and tables. KA, RP and KK obtained funding. All authors provided critical review and contribution to the draft.

## CRediT authorship contribution statement

**Md Hamidul Huque:** Writing – review & editing, Methodology, Formal analysis. **Ranmalee Eramudugolla:** Visualization, Formal analysis, Data curation. **Meiwei Li:** Visualization, Data curation. **Kim M. Kiely:** Funding acquisition. **Ruth Peters:** Funding acquisition. **Kaarin J. Anstey:** Writing – original draft, Supervision, Investigation, Funding acquisition, Conceptualization.

## Declaration of competing interest

The authors declare that they have no known competing financial interests or personal relationships that could have appeared to influence the work reported in this paper.

## Appendix Supplementary materials

Image, application 1
