# Supplementary material for: A critical review and classification of dementia risk assessment tools to inform dementia risk reduction
Source: J Prev Alzheimers Dis. 2025 Sep 2;12(9):100333. doi: 10.1016/j.tjpad.2025.100333 (PMC12501348; doi:10.1016/j.tjpad.2025.100333)
Supplement: Supplementary file 1 [file mmc1.docx]

**Supplementary materials**

**Contents:**

**Table S1** Characteristics of all cause dementia risk tools according to the development cohorts and outcome

**Table S2** Characteristics of all the validation studies considered in this review

**Table S3** AD Risk factors considered in developing AD risk scores.

**Table S4.** Prediction model Risk of Bias Assessment for all the studies considered in this review.

**Table S5** Estimated relative costs and health services required for administering each Dementia Risk Tool in a clinical or community setting.

**Figure S1** Pooled c-statistics of the developed and validation of midlife (left panel) and midlife to late-life dementia risk assessment tools

**Figure S2** Pooled c-statistics of the developed and validation of community-based late-life (left panel) and clinical dementia risk assessment tools

**Figure S3** Pooled c-statistics of the widely validated dementia risk assessment tools.

**Figure S4** c-statistics dementia risk scores with various predictors

**Figure S5** Pooled c-statistics of the development and validation of AD risk assessment tools.

**Figure S6** Pooled c-statistics of the widely validated AD risk assessment tools.

**References** for studies selected for tools identification and quantitative synthesis.

**Supplementary Material: PICO Framework**

We used following PICO framework (Population, Intervention, Comparison, Outcome) to define eligibility criteria.

- **Population (P):** We included studies involving participants aged 18 years or older who were free from diagnosed dementia at baseline. Eligible populations included (i) cognitively normal adults, (ii) individuals with SCD, and/or (iii) those with MCI. These populations are commonly targeted in dementia prevention and risk assessment. Studies with mixed populations were included if baseline dementia was excluded, and subgroup composition was reported.
- **Intervention / Index Tool (I):** Multivariable risk prediction models or scoring tools estimating future dementia or cognitive decline risk, using any combination of predictors (e.g., demographic, clinical, genetic, lifestyle, cognitive), regardless of setting.
- **Comparison (C):** No explicit comparator was required. However, studies that compared multiple tools, or the same tool across different populations, settings, or time periods were included.
- **Outcome (O):** Incident dementia diagnosed via clinical criteria, ICD codes, or validated algorithms. Cognitive decline outcomes were included if clearly defined and relevant to dementia risk (e.g., MCI conversion or validated cognitive thresholds).

**Table S1.** **Characteristics of all cause dementia risk tools according to the development cohorts and outcome**

| Paper details | | | Sample details | | | | Methodological details | | | | Risk score details | | Results |
| --- | --- | --- | --- | --- | --- | --- | --- | --- | --- | --- | --- | --- | --- |
| First author, year | **Risk tool** | **Tool ID** | **Settings, country** | **Sample  size** | **% male** | **Age range** | **Method of dementia diagnosis** | **Main**  **Outcome for risk tool** | **Total follow-up length** | **Duration of incidence predictions** | **Risk categories and thresholds used** | **Variables used in model** | **AUC/C-statistics /Risk ratios** |
| Risk score developed using midlife risk factor information | | | | | | | | | | | | | |
| Eskelinen 2011[1] | CAIDE midlife healthy-diet index | 5 | Community sample, Finland | 385 | 38% | Baseline:  Low adherence (0–8 points): 56.9;  High adherence (>8 points): 57.2; Follow-up: 65 -79 | DSM-IV for dementia; NINCDS-ADRDA for AD | Dementia | 14 Yrs | None | Total score ranging from 0 (minimal adherence to healthy  diet) to 17 (maximal adherence) with a cutoff score of > 8 point | Beneficial components *(e.g., vegetables and roots);* Unhealthy components *(e.g., sausage foods);* Alcohol drinking; Use of salt; Type of fats used for cooking | **Odds ratio (95% CI)**: For AD: 0.10 (0.01–0.94) For dementia: 0.14 (0.02–0.80) |
| Kivipelto 2006[2] | CAIDE risk score | 6 | CAIDE study, Finland | 1,409 | 38% | 39-64 Yrs old at baseline (mean age =50.4); 65-80 Yrs old at follow up  (mean age = 71.3) | DSM-IV for dementia; NINCDS-ADRDA for AD | Dementia | 20.9 Yrs | None | Sum score range 0–18 with cut-off of 10 points | Age, sex, education, SBP, BMI, total cholesterol, physical activity, Apoe ε4. | **AUC (95% CI):**  **Without Apoe ε4**:  0.77 (0.71, 0.83)  **With Apoe ε4**: 0.78 (0.72–0.84) |
| Ibarrondo 2022[3] | Risk scores to predict the late-life risk of dementia | 27 | Community population, Spain | 25,015 | 57% | 30 – 70.  Age at recruitment: 49.08 | ICD-9, ICD-10 | Dementia | 25 Yrs | None | For entire population: low < 8, moderate 8–11, and high > 11 For those ≥ 55 Yrs: low < 12, moderate 12–16, and high > 16 | Age, sex, physical activity, hypertension, hyperlipemia, diabetes, Cambridge Index, smoking, education, BMI, diet | **AUCs:** Overall: 0.603 (0.533-0.661) For over 55 year olds: 0.55 (0.48-0.61) |
| Liang 2020[4] | Cardiovascular health (CVH) metrics | 7 | CAIDE study, Finland | 1,449 | 38% | mean age 50.4 Yrs | DSM-IV | Dementia | 8.3 Yrs and 21.2 Yrs | None | Possible range: 0 - 14 Poor level: 0 to 5 intermediate: 6 to 7 Ideal: ≥8 | Smoking, physical activity, BMI, fasting plasma glucose,  total cholesterol, serum total cholesterol and blood pressure | **Hazard ratio (HR)**   0.71 (95% CI: 0.43, 1.16) |
| Vuoksimaa 2016[5] | Education and occupation-based  middle age self-report risk score | 15 | Finnish Twin Cohort (FTC) study, Finland | 2,602 | 52% | Baseline:  38.4 - 69.5  Mean age: 47 years. Follow-up Mean age: 74.4 | Screening tools | Dementia | 23 to 31 Yrs | None | Possible range:  Clinical use: 3 - 25 (with age) with a cutoff score <16 Research use: 0 - 19 (without age) with a cutoff score <12 | Age, education, working status, nature of work, work environment, physicality of work | **AUC (95% CI):** 0.77 (0.74–0.80) |
| Jung et al., 2024[6] | Midlife-Primary Care risk score | 42 | Korean National Health Insurance Service database | 3,248,171 | 51% | Age 40-59  (Mean age: 48.5) | ICD 10 code F00, F01, F02, F03, G30, G23.1, G31) | Dementia | 8.95 years | 5-year risk | Possible ranges: 0 to 243 | Age, sex,  obesity, smoking, alcohol consumption, physical activity, diabetes, hypertension, dyslipidaemia, and  Chronic kidney disease. | **N/A** |
| Tools developed using midlife to late-life risk factor information (cohorts data analysis) | | | | | | | | | | | | | |
| Wang 2022b[7] | Modified dementia risk scores(MDRS) | 24 | UK Biobank, UK | 239,745 | 53% | ≥40 | ICD-9 | Dementia | 8.7 Yrs | 14-year risk | Possible range: Model 1: 0 - 154 with a cutoff score of 81; Model 2 (including the APOEε4): 0 - 180 with a cutoff score of 98 | Age, education, sex, physical activity, smoking, glycaemic status, depressive symptoms, APOE ε4 | **AUCs:** Model 1: 0.81 (95% CI: 0.804 - 0.816) Model 2: 0.832 (95% CI: 0.826–0.838) |
| Rawtaer 2016[8] | Neurocognitive Disorders Risk Score | 25 | Singapore Longitudinal Ageing Study (SLAS) 1 | 957 | 38% | >55 | Screening tools | Dementia | 5 Yrs | None | Possible range 0 to 151 with a cutoff score of 45 | Age, gender, education, depression, heart disease, social and productive activities and MMSE score | **AUC (95% CI):** 0.72 (0.66–0.77) |
| Tolea 2021[9] | mCAIDE risk score | 21 | Community sample, USA | 219 | 40% | ≥ 40 | Screening tool;  Neuropsychological evaluation | Cognitive impairment | N/A | None | Possible range 0 to 14 Low risk: 0 Moderate risk: 7 High risk: 14 | Age, sex, education, SBP, BMI, self-report high cholesterol, mini Physical Performance Testing (PPT) | **AUC (95%CI):**  0.634 (0.548–0.721) |
| Ren 2022† [10] | Clinical Dementia Risk Score Prediction Tool | 9 | UK Biobank, UK | 444,695 | 46% | ≥40 | ICD-10 | Dementia | 13 Yrs | 5-, 9-, and 13-year risk | Total point score of the risk score model ranges from −18 to 30 in men and −17 to 30 in women | Age, educational level, body mass index, smoking, frequency of alcohol consumption, employment status, sleep, respiratory disease, cancer, cerebrovascular disease, diabetes, cardiovascular disease, and hypertension | **C-statistic:**  training data set: 0.86 for men and 0.85 for women ; |
| Ng 2021† [11] | SLAS Risk Index | 32 | Singapore Longitudinal Ageing Study (SLAS) I, Singapoure | 1610 | 34% | ≥55 | DSM-4R | MCI/Dementia | 4.5 Yrs | None | Possible range: 0-12 with a cutoff score of 6 | Age, female sex, Yrs of schooling, hearing loss, depression, life satisfaction, number of cardio-metabolic risk factors | **AUC (95%CI):** 0.73 (0.70, 0.75) |
| Anatürk et al., 2023[12] | UKBDRS | 36 | UK Biobank, UK | 223,696 | 51% | UKB: 40-73 (Mean age: 60)  WHII: 35-55 (Mean age: 57) | Clinical diagnosis/criteria | Dementia | 14 years | 14-year risk | NA | Age, education, parental history of dementia, material deprivation, a history of diabetes, stroke, depression, hypertension, high cholesterol, household occupancy, and sex | **AUCs:**  UKB: 0.8 (95% CI: 0.78 - 0.82) |
| Tools developed using midlife to late-life risk factor information (Evidence synthesis) | | | | | | | | | | | | | |
| Anstey 2013† [13] | ANU-ADRI | 3 | Evidence synthesis | N/A | N/A | ≥40 years | N/A | AD | N/A | None | Possible range: -18 to 76 | Age, sex, education, obesity, diabetes, depression, high cholesterol, TBI, smoking, alcohol consumption, social engagement, physical activity, cognitive activity, fish intake, pesticide exposure | N/A |
| Deckers et al 2015† [14] | LIBRA risk score | 20 | Evidence synthesis and Delphi method | N/A | N/A | ≥45 years | N/A | Dementia | N/A | N/A | Possible range: -5.9 to 12.7 | Depression, midlife obesity, midlife hypertension, physical inactivity, diabetes, depression, hyperlipidaemia, smoking, coronary heart disease, renal dysfunction, MIND diet and cognitive activity. | **N/A** |
| Anstey 2022† [15] | Cognitive Health and Dementia  Risk Reduction (CogDrisk) | 12 | Evidence synthesis | N/A | N/A | ±40 | N/A | Dementia | N/A | None | Possible ranges: -4.25 to 44 | Age, sex, education, midlife obesity, high cholesterol, diabetes, stroke, TBI, hypertension, Atrial fibrillation, insomnia, depression, physical activity, cognitive activity, social engagement, fish intake, smoking | **N/A** |
| Anstey 2022† [15] | CogDrisk-AD | 39 | Evidence synthesis | N/A | N/A | ≥40 | N/A | AD | N/A | None | Possible ranges: -3.4 to 42 | Age, sex, education, midlife obesity, high cholesterol, diabetes, stroke, TBI, hypertension, depression, physical activity, cognitive activity, social engagement, fish intake, smoking, pesticide exposure | **N/A** |
| Tools developed using Late-life risk factor information using community sample | | | | | | | | | | | | | |
| Barnes 2014a[16] | Brief Dementia Screening Indicator (BDSI) | 4 | The Cardiovascular Health Study (CHS); Framingham Heart  Study (FHS);  the Health and Retirement Study (HRS);  the Sacramento Area Latino Study on  Aging (SALSA), USA | CHS (n = 2794); FHS (n = 2411); HRS (n = 13,889); SALSA (n = 1125) | CHS: 40.4% FHS: 44.5% HRS: 43.5% SALSA: 42.8% | 65-79 | DSM-IV; Screening tool | Dementia | 10 - 20 Yrs | None | Total score ranging from 0 to 56 points with a cut point of ≥ 22 | Age, education, stroke, diabetes, BMI, depressive symptoms, assistance needed with money or medications | **C statistic (95% Cl):**  **CHS**: 0.68 (0.65–0.72) **FHS**: 0.77 (0.73–0.82) **HRS**: 0.76 (0.74–0.77) **SALSA**: 0.78 (0.72–0.83) |
| Lin 2018[17] | Japan Gerontological Evaluation Study (JAGES) risk index | 18 | Japan Gerontological Evaluation Study (JAGES), JAPAN | 72,127 | 44% | ≥65 | Long-term care (LTC)  certification | Dementia | 3 yrs & 4 month | None | Model 1: Maximum risk score 10 with the cut-off values 5/6 Model 2: Maximum risk score 59 with the cut-off values 23/24 Model 3: Maximum risk score 62 with the cut-off values 26/27 | Gender, IADL, physical function, nutritional status, housebound ness, cognitive function, health checkup, BMI, fasting blood sugar level | **C-statistic (95% CI)** Model 1: 0.733 (0.726–0.739) Model 2: 0.790 (0.784–0.796) Model 3: 0.786 (0.780–0.792) |
| Reitz 2010† [18] | Late-onset Alzheimer's disease (LOAD) Risk Score | 19 | Community population, USA | 1,051 | 34% | ≥65 | DSM-IV for dementia; NINCDS-ADRDA for late-onset AD | AD | 4 yrs | None | Possible range: 0 -60 A higher score indicating higher risk | Age, sex, diabetes, hypertension, smoking, low HDL-C, high WHR, education, ethnicity, APOE ε4 | **N/A** |
| Downer 2016[19] | Mexican-American Dementia Nomogram (MADeN) | 22 | Hispanic Established Populations for the Epidemiologic Study of the Elderly (H-EPESE) , USA | 1,739 | 42% | ≥65 | Alzheimer’s Association and National Institute on Aging Workgroup proposed guidelines | Dementia | 11 Yrs | 10-year risk | Possible range: 0 - 28+ depending on age A higher score indicating higher risk | Age, sex, education, social engagement, diabetes mellitus, feeling the blues, pain, IADL, and unable to walk a half-mile | **ROC (95% CI):** 0.74 (0.70–0.78) |
| Capuano 2022[20] | Rapid Assessment of Dementia Risk (RADaR) | 26 | Rush Memory and Aging Study (MAP); Religious Order Study (ROS); Minority Aging Research Study (MARS), USA | MAP: 1,780 ROS: 1,299 MARS: 679 | 25% | mean age at baseline  76.8 | NINCDS-ADRDA | Dementia | 10 Yrs | 3-year risk | Age: Add 5 points for every year above 65 Function status: 0 -70 Memory complaint: 0-30 Orientation: 0 -180 Delayed recall: Add 50 for each word didn't remember | Age, memory complaint, the ability to handle finances, the recall of the month, recall of the room, and recall of three words | AUC: 0.82–0.86 |
| Licher 2017[21] | Rotterdam Study Basic Dementia Risk Model (BDRM) | 29 | Rotterdam Study, Netherlands | 2,710 | 47% | >60 | DSM-III-R for dementia; NINCDS-ADRDA for AD | Dementia/AD | Median: 7 Yrs | 10-year risk | A higher score indicating higher risk | Age, history of symptomatic stroke, presence of subjective memory decline, need for assistance with finances or medication, cognitive testing, APOE-ε4 carrier status, brain MRI parameters | **C statistic (95% CI):**  **For dementia:** Basic model: 0.78 (0.75, 0.81) Extended model: 0.86 (0.83, 0.88)  **For AD:**  Basic model: 0.77 (0.75, 0.81) Extended model: 0.86 (0.83, 0.88) |
| Makino 2021† [22] | Simplifed Telephone Assessment for Dementia risk (STAD) | 31 | Community population, Japan | 4,298 | 50% | ≥65 | ICD-10 | Dementia | 24 months | None | Total score ranging from 0 to 12 with a cutoff score of ≥5 | Subjective cognitive complaints, depressive symptoms, and lifestyle activities | **Hazard ratio (95% CI):** 2.67 (1.40–5.08) |
| Barnes 2009[23] | The late-life dementia risk index | 34 | Cardiovascular Health Cognition Study, USA | 3,375 | 41% | ≥65  mean age =76 Yrs old | NINCDS-ADRDA for AD; ADDTC for VaD | Dementia/AD | 6 Yrs | None | Possible range 0 to 15 Low risk: 0 -3 Moderate risk: 4-7 High risk: ≥8 | Older age; Poor cognitive test performance; BMI, APOE ε4; Cerebral MRI findings of white matter disease; Ventricular enlargement; Internal carotid artery thickening on ultrasound; History of bypass surgery; Slow physical performance; lack of alcohol consumption | **AUC (95%CI):**  **Dementia:** 0.81 (0.79, 0.83)  **AD:** 0.79 (0.76, 0.82) |
| Santabárbara 2021† [24] | ZARADEMP Alzheimer Dementia Risk Score | 35 | Zaragoza Dementia and Depression (ZARADEMP) Project, SPain | 3,044 | 44% | ≥65 | DSM-IV | AD | 4.4 Yrs | 5-year risk | Possible range 0 to 56 For each one-point increment, the AD risk increased by 16% | Age, sex, education, marital status, depression, anxiety, BMI, hearing loss | **SHR (95% CI):** 1.16 (1.12–1.19) |
| Walters 2016† [25] | Routinely collected health data algorithm | 37 | Health Improvement Network (THIN) database, UK | 930,395 | 48% | 60-95 (Mean age: 65.6) | ICD-10 | Dementia | <5 Yrs | 5-year risk | A higher score indicating higher risk | Age, sex, social deprivation, smoking, BMI, heavy alcohol use, anti-hypertensive drugs, diabetes, stroke/TIA, atrial fibrillation, aspirin, depression | **N/A** |
| Huque 2024[26] | DemNCD | 40 | Atherosclerosis Risk in Communities  (ARIC);  CHS-CS; HRS ADAMS;  MAP,  FHS study, the  MRC  CFAS-I and CFAS-II), the Sydney Memory and  Aging Study (MAS), the Maastricht Aging Study  (MAAS)  and  the Singapore Longitudinal Ageing Study-I (SLAS-I) . | 27,162 | 41.6% | ≥65 | DSM-III-R, IV criteria or ICD codes. Algorithms were used for diagnosis of dementia in ARIC and CFAS. | Dementia | Mean (SD): 7.1 (5.5) | None | Range: -34, 44, A higher score indicating higher risk | Age, sex, education, obesity, smoking, alcohol consumption. high cholesterol, HDL, LDL, hypertension, diabetes, stroke, TBI, depression, physical activity, cognitive activity, loneliness, fish intake, fruits & vegetable intake, Atrial Fibrillation, sleep problem, hearing loss | **RoC**  0.68 (0.67, 0.70) |
| Chen 2024[27] | Polysocial risk score | 41 | HRS study | 5199 | 42% | ≥60 | Langa-Weir dementia classification Algorithm | Dementia | 6.2 years | None | 0 to 10 | Age, sex, education, race, depression, hypertension, diabetes, hearing impairment, Economic stability, health care access and equity, Neighbourhood and built environment, region, marital status, religious activity, living arrangements, social support, social cohesion, lifetime stressful event, and discrimination. | **AUC:** 0.78 (0.77, 0.79) |
| Jung et al., 2024[6] | Late-life Primary Care risk score | 43 | Korean National Health Insurance Service database | 1,439,081 | 47% | Age ≥60  (Mean age: 66.7) | ICD 10 code F00, F01, F02, F03, G30, G23.1, G31) | Dementia | 6-years | 5-year risk | Possible ranges: 0 to 174 | Age, sex,  obesity, smoking, alcohol consumption, physical activity, diabetes, hypertension, dyslipidaemia, and  Chronic kidney disease. | **N/A** |
| Pan 2024[28] | FDRS-DMSV | 44 | AIBL, Australia | 1407 | 43% | 77.7 | Not given | AD | NA | NA | Possible range: 0 to 100 | Age, sex, depression, Diastolic and Systolic blood pressure, Heart rate, Neurological disorder, Present living arrangements, Primary occupation, difficulty with memory, Marital status, Arthrities, Anxiety, Hypertension, recent illness | **AUC: 0.78 (0.73-0.84)** |
| Tools developed using risk factor information using clinical sample | | | | | | | | | | | | | |
| Jessen 2011 [29] | AgeCoDe | 1 | Aging, Cognition and Dementia study (AgeCoDe), Germany | 3,055 | 35% | Age ≥75 | DSM-IV for dementia; NINCDS-ADRDA for AD; NINDS-AIREN for VaD | AD | 18 months | None | Possible range: 0 to 21  Low risk: 0 -8 Moderate risk: 9-14 High risk: ≥15 | Age, subjective memory impairment, verbal flunecy, delayed recall, MMSE, IADL | **AUCs and 95% CI:** First cohort: 0.84 (0.80–0.88); Test cohort: 0.79 (0.74–0.84) |
| Spinar et al. 2016†† [30, 31] | AHEAD scores | 2 | GREAT registry, Czech republic | 5846 | 43.4% | 51-88 |  | CVD: Atrial fibrillation (AF) |  |  |  | Age, sex, BMI, AF, Diabetes, Hypertension, COPD, acute coronary syndrome, Chronic Ischemic heart disease, Creatinine, b;lood Natrium level, Anemia | **N/A** |
| Camm et a. 2013†† [30, 32] | CHA_2_DS­_2_ -VASC scores | 8 | 2012 focused update of the ESC Guidelines for the management of atrial fibrillation |  |  | >65 years |  | CVD: Atrial fibrillation |  |  |  | Age, sex, Ischaemic stroke, Intracranial bleeding, Myocardial Infraction, CABG, Peripheral artery disease, hypertension, heart failure, diabetes, thyroid, thyrotoxicosis | **N/A** |
| Lee 2014[33] | Clinical index to predict conversion from  amnestic MCI to probable Alzheimer’s disease | 10 | Alzheimer's Disease Neuroimaging Initiative (ADNI) , USA | 382 | 64% | 55–90 | NINCDS-ADRDA for AD | AD | 36 months | 3-year risk | Possible range: 0-16 Low risk: 0-2 Moderate risk: 3-8 High risk: 9-16 | Gender, functional dependence, neuropsychiatrist symptoms, cognitive measures | **C-statistic 95% CI:**  0.71(0.68–0.75) |
| Dharmasaroja 2022[34] | Clinical risk score for vascular dementia  after ischemic stroke (HASTE) | 11 | Hospitalised for Acute ischaemic stroke, Thailand | 177 | 67% | Non-dementia: Mean age = 61.7 (SD=11.2) Vascular dementia: Mean age = 74.5 (SD=9.6) | NINDS-AIREN for VaD | VaD | 6 months | None | Score ranges 0-11 points with a cutoff score of ≥5 | Age, education, history of stroke, white matter hyper-intense lesions, stroke subtype | **AUROC (95% CI):** 0.76 (0.69, 0.83) |
| Li 2018a [35] | Dementia risk prediction model for  Chinese type 2 diabetes patients | 13 | Diagnosed with Diabetes and in registry, Taiwan | 27,540 | 45% | 50–94 | ICD-9-CM | Dementia | Ave: 8.09 Yrs | 3-, 5- and 10- year | Possible range: 0 to 20 A higher score indicating higher risk | Age, sex, duration of type 2 diabetes, BMI, variation in FPG, and HbA1c, stroke, hypoglycemia, postural hypotension, coronary artery disease and anti-diabetes medication | **AUCs and 95% CI:** 3-year: 0.82 (0.80, 0.84),  5-year: 0.79 (0.77, 0.81)  10-year: 0.76 (0.75, 0.77) |
| Exalto 2013† [36] | The dialysis dementia risk score (DSDRS) | 14 | Algorithmically classified Diabetes  based on records and in registry, USA | 29,961 | 53% | ≥60 | ICD-9-CM for dementia; ICD 9 for AD and VaD | Dementia | 10 Yrs | None | Possible range: −1 to ≥12 | Age, education, microvascular disease, diabetic foot, cerebrovascular disease, cardiovascular disease, acute metabolic event, depression | **C-statistic:**  Diabetes and Aging Study: 0.733 |
| Wilson 1998†† [37] | The Framingham risk score | 16 | Framingham heart study, USA | 5345 | 54% | 30-74 | N/A | CVD | 10 Yrs | NA | NA | Age, hypertension, smoking, diabetes, LDL, HDL | **N/A** |
| Graves 2018† [38] | Intermountain Mortality Risk Score | 17 | Diagnosed with atrial fibrillation, USA | 74,081 | 54% | ≥18 | ICD-9 and ICD-10 | CVD | 5 Yrs | None | NR | Age, sex, admission complete blood count and basic metabolic profile | N/A |
| Wang 2022a[39] | Mild Cognitive Impairment to Dementia Risk (CIDER) score | 23 | Diagnoses with MCI, USA and Canada | 6,222 | 50% | ≥55 | DSM-IV | Dementia | Within 3 Yrs | 3-year risk | Possible range: 0 to 155 (MoCA model) Possible range: 0 to 160 (MMSE model) A higher score indicating higher risk | Age, sex, education, marital status, MMoCA or MMSE score, hypertension, mood disorder | **C-statistic (95% CI):** CIDER MoCA: 0.69 (0.66, 0.73) CIDER MMSE: 0.67 (0.65, 0.69) |
| Barnes 2014b [40] | Risk tool to predict conversion from  amnestic MCI to probable AD | 28 | Alzheimer's Disease Neuroimaging Initiative (ADNI), USA and Canada | 382 | 64% | 55–90 Yrs | NINCDS-ADRDA for AD | AD | 36 months | 1- year and 3- year risk | Possible range 0 to 9  Low risk: 0-3 Moderate risk: 4-6 High risk: 7-9 | Functional Assessment Questionnaire; MRI middle temporal cortical thinning; MRI hippocampal subcortical volume; Alzheimer’s Disease Cognitive Scale; and the Clock Test | **C-statistic 95% CI:** 0.78 (0.75, 0.81) |
| Mehta 2016[41] | RxDxDementia Risk Index | 30 | Clinical Practice Research Datalink (CPRD), UK | 133,176 | 52% | ≥60 | Clinical diagnosis and prescription medication use | Dementia | 9 Yrs | None | Possible range from –9 to 29 | Age, gender, and 31 RxDx disease conditions (e.g., myocardial infarction, epilepsy, depression) | **C-statistic 95% CI:** 0.806 (0.799–0.812) |
| Ling 2022[42] | The dialysis dementia risk score (DDRS) | 33 | Patients with kidney failure, Taiwan | 32,188 | 44% | 48-80 | ICD-9 | Dementia | 12 Yrs | None | Possible range 0 to 107 with a cutoff score of 50 | Age, diabetes mellitus, stroke, anemia, hypertension, hyperlipidemia, depression, insomnia, TBI, Parkinson's disease, hypothyroidism | **C statistic** with 95% CI:  0.71 (0.70–0.72) |
| Hou 2023[43] | Multipredictor risk models for AD | 38 | ADNI, USA and Canada | 1,283 | 55% | 74.8 | Clinical diagnosis/criteria | AD | 4.5 Yrs - 5.6 Yrs | 5-year risk | **CN risk model:** Possible ranges from 0 to 32 with a higher score indicating higher risk; | **CN risk model:** Age, depression, diabetes, SBP, Alzheimer’s Disease Assessment Scale with 11 items (ASAS11), MMSE, Rey's Auditory Verbal Learning Test (RAVLT), Functional Assessment Questionnaire (FAQ), Logical memory delayed recall (LM-DR); | **AUCs:**  CN risk model: 0.75 (95% CI 0.69–0.82)  **MCI risk model:** 0.88 (95% CI 0.85–0.91) |
| Pan 2024[28] | Florey Dementia Risk Score (FDRS) | 45 | AIBL, Australia | 1407 | 43% | 77.7 | Not given | AD | NA | NA | Possible range: 0 to 100 | Age, geriatric depression, diastolic blood pressure, Systolic blood pressure, Heart rate, APOE, Neurological disorder, Present living arrangements, Primary occupation, difficulty with memory, Marital status | **AUC: 0.82 (0.75-0.88)** |

†: Not included in quantitative synthesis, ††: Original papers that developed risk tools for CVD risk prediction.

**Table S2.** **Characteristics of all the validation studies considered in this review**

| Tool details | | | | Validation study sample details | | | | Methodological details | | Risk score details | Results |
| --- | --- | --- | --- | --- | --- | --- | --- | --- | --- | --- | --- |
| Tool ID | **Risk tool** | **Development sample** | **Outcome** | **First author, year** | **Settings, country** | **Sample  size** | **% male** | **Outcome for risk tool**  **(diagnosis method)** | **Total follow-up length** | **Variables used in model** | **AUC/C-statistics (95% CI)** |
| Validation of tools that were developed using midlife risk factors information | | | | | | | | | | | |
| 6 | CAIDE | **Community**  Age 39-64 years | **Dementia** | **Exalto et al 2024[44]** | Kaiser Permanente members, USA  Age: 40-55 | 9,480 | 45% | Dementia | 12 Yrs | CAIDE (minus physical activity) | **C statistic:** 0.75 |
|  |  |  |  | **Chosy et al 2019 [45]** | Honolulu Heart Program, USA  (Avg) age=53.1 Yrs; | 3,582 | 100% | Dementia | 20 Yrs | CAIDE (minus APOE ε4 status) | **C-statistic (95%CI):** 0.645 (0.62– 0.67) |
|  |  |  |  | **Stephan et al 2020 [46]** | 10/66 Study  Age≥65 | 11,143 | 37% | Dementia,  (10/66 diagnostic  algorithm) | Avg. 3.8 years | Age, sex, education, systolic blood pressure,  BMI, total cholesterol, and physical activity | **C-statistic (95%CI):** 0.55 (0·53–0·57) |
|  |  |  |  | **Tolea et al 2021 [9]** | Clinical sample,  Age: 74.34±9.84, South Florida academic dementia center, USA | 219 | 50.2% | MCI or dementia | N/A | Age, sex, education, BMI, SBP, high cholesterol, mini-PPT, | **C-statistic (95%CI):**0.709 (0.643, 0.803) |
|  |  |  |  | **Huque et al 2023 [47]** | HRS ADAMS;  Age>70years  CHS-CS;  Age≥65  MAP,  Age >60 years,  USA. | HRS ADAMS (n = 856);  CHS-CS (n = 3602);  MAP (n = 2184) | HRS ADAMS: 47.5%  CHS-CS: 40.9%  MAP: 26.5% | Dementia | >5 Yrs | age, gender,  education, obesity, traumatic brain injury, physical activity, and hypertension | **C statistic**  HRS-ADAMS: 0.56 (0.49, 0.63)  CHS-CS cohort:  0.57 (0.52, 0.61)  MAP:0.50 (0.46-0.54) |
|  |  |  |  | **Anatürk et al 2023 [12]** | UK Biobank,  Age: 50-73  Whitehall II study,  Age: 50- 67 (Mean age: 57)  UK | UK Biobank, n=44151,  WHII, n=2934 | UK Biobank, 49.5%  Whitehall II study, 71.6% | Dementia | 14 years | Age, education, obesity, diabetes, stroke, depression, hypertension, high cholesterol, physical activity and fish intake | **C statistic**  UKB:0.60 (0.58, 0.63)  Whitehall: 0.69 (0.64, 0.74) |
| 15 | Education and occupation-based middle age self-report risk score | **Community sample,**  **Age:** 38.4-69.5 | Dementia | **Vuoksimaa et al. 2016 [5]** | Finnish Twin Cohort (FTC) study, Finland  Age: 38.4-69.5 | 2,602 | 52% | Dementia, TICS | Average 39.1 years | Age, education, working status, nature of work, work environment, physicality of work | **C statistic**  **0.72 (0.65, 0.79)** |
| 42 | Midlife- Primary Care risk score | **Community sample. Age: 40-59 years** | **Dementia** | **Jung et al., 2024[6]** | Korean National Health Insurance Service database; Mean age: 66.7 | 1,391,977 | 51% | Dementia | 9 years | Age, sex,  obesity, smoking, alcohol consumption, physical activity, diabetes, hypertension, dyslipidaemia, and  Chronic kidney disease. | **AUC:** 0.764 (0.760, 0.768) |
| Validation of tools that were developed using midlife to late life risk factors information (Cohort data) | | | | | | | | | | | |
| 9 | Clinical Dementia Risk Score Prediction Tool | **Community sample**  Age: 40-73 | **Dementia** | **Ren 2022 [10]** | UK Biobank, UK  Age: 40-73 | 444,695 | 46% | Dementia | 13 Years | Age, educational level, body mass index, smoking, frequency of alcohol consumption, employment status, sleep, respiratory disease, cancer, cerebrovascular disease, diabetes, cardiovascular disease, and hypertension | **C statistic**  Testing data set: 0.85 for men and 0.87 for women |
| 21 | mCAIDE risk score | **Community sample** | **MCI/Dementia** | **Tolea 2021 [9]** | Clinical sample, USA  Age: 74.3±9.8 | 219 | 40% | MCI or Dementia | Cross-sectional | Age, sex, education, SBP, BMI, self-report high cholesterol, mini Physical Performance Testing (PPT) | **AUC**  0.78 (0.71, 0.85) |
| 32 | SLAS Risk score | **Community sample** | **MCI or dementia** | **Ng 2021 [11]** | Singapore Longitudinal Ageing Study (SLAS) II, Singapore.  Avg age: 65 | 1323 | 35.7% | MC or dementia | 4.5 Years | Age, female sex, Yrs of schooling, hearing loss, depression, life satisfaction, number of cardio-metabolic risk factors | **AUC**  0.74 (0.72, 0.77) |
| 36 | UKB risk score | **Community sample.** | **Dementia** | **Anatürk et al 2023 [12]** | UK Biobank (UKB),  Age: 50-73  Whitehall II study,  Age: 50- 67 (Mean age: 57)  UK | UKB, n=44151,  WHII, n=2934 | UKB, 49.5%  Whitehall II study, 71.6% | Dementia | 14 years | Age, education, obesity, diabetes, stroke, depression, hypertension, high cholesterol, physical activity and fish intake | **C statistic**  UKB:0.80 (0.78, 0.82)  Whitehall: 0.77 (0.72, 0.81) |
| Validation of tools that were developed using midlife to late life risk factors information (Evidence synthesis) | | | | | | | | | | | |
| 3 | ANU-ADRI | **Community**  **Age >65 years** | **AD** | **Anstey et al. 2014 [48]** | Rush Memory and Aging Study (MAP); Kungsholmen Project (KP); Cardiovascular Health Cognition Study (CVHS) | MAP: 1,146 KP: 1,301 CVHS: 5,201 | N/A | Dementia (DSM-III-R ) | MAP: 3.5 Yrs KP: 6 Yrs CVHS: 6 Yrs | **MAP:** ANU-ADRI (minus fish intake and depression); **KP:** ANU-ADRI (minus physical activity, fish intake and depression); **CVHS**: ANU-ADRI (minus TBI, cognitive activity and social engagement) | **MAP study**: 0.637 (0.596–0.678)  **KP study**: 0.740 (0.712–0.768)  **CVHS study**:  0.733 (0.691–0.776) |
|  |  |  |  | **Stephan et al. 2020 [46]** | 10/66 Study  Age≥65 | 11,143 | 37% | Dementia  (10/66 diagnostic  algorithm) | Avg. 3.8 years | Age, sex, education, height-to-weight ratio, diabetes, depression total cholesterol, smoking, alcohol use, traumatic brain injury, physical activity, social engagement, and fish intake | 0.69 (0.67, 0.71) |
|  |  |  |  | **Huque et al 2023 [47]** | HRS ADAMS;  Age>70years  CHS-CS;  Age≥65  MAP,  Age >60 years,  USA.. | HRS ADAMS (n = 856);  CHS-CS (n = 3602);  MAP (n = 2184) | HRS ADAMS: 47.5%  CHS-CS: 40.9%  MAP: 26.5% | Dementia,  AD | >5 Yrs | Age, sex, education, high cholesterol, stroke, TBI, hypertension, atrial fibrillation, insomnia, depression, physical inactivity, cognitive engagement, social engagement, diet, smoking | **For dementia:**  HRS-ADAMS: 0.66 (0.59, 0.73)  CHS-CS cohort:  0.69 (0.66, 0.72)  MAP:0.65 (0.61-0.69)  **For AD:**  HRS-ADAMS: 0.66 (0.58, 0.75)  CHS-CS cohort:  0.71 (0.68, 0.74)  MAP:0.65 (0.61-0.69) |
| 12 | CogDrisk tool | **Community sample.**  **Age>65** | **Dementia** | **Huque et al 2023 [47]** | HRS ADAMS;  Age>70years  CHS-CS;  Age≥65  MAP,  Age >60 years,  USA. | HRS ADAMS (n = 856);  CHS-CS (n = 3602);  MAP (n = 2184) | HRS ADAMS: 47.5%  CHS-CS: 40.9%  MAP: 26.5% | Dementia | >5 Yrs | Age, gender, education, obesity, diabetes, depression, traumatic brain  injury, smoking, loneliness, physical activity, cognitive activity, fish intake, stroke, and  hypertension; | **C statistic**  HRS-ADAMS: 0.65(0.58, 0.72)  CHS-CS cohort:  0.70 (0.67, 0.72)  MAP:0.65 (0.61-0.69) |
|  |  |  |  | **Kootar et al 2023 [49]** | SNACK-K,  Age: >60 years  Sweden | N=2943 | 36.6% | Dementia | 8 Years | Age, Gender, Education, Diabetes, Stroke, Hypertension, Smoking, TBI, Atrial fibrillation, Insomnia, Depression, Loneliness, Physical activity, Cognitive activities and Fish intake | **C statistic**  0.77 (0.57, 0.97) |
| 20 | LIBRA risk score | **Community sample**  **Age≥40** | Dementia | **Vos et al 2017 [50]** | DESCRIPAstudy,  Age: 55-69y (midlife) and 70-79 (latelife) | Midlife, n=3256  Late-life, n=4320 | 45% | Dementia | Midlife: 8.1 years  Late-life: 7.2 years | Age, sex, education, Depression, Hypertension, Obesity, Smoking, Hypercholesterolemia, Diabetes, Physical activity, Coronary heart disease, Alcohol consumption. | **C statistic**  Midlife 0.57 se 0.03  Late-life: 0.50 se 0.01 |
|  |  |  |  | **Schiepers et al 2018 [51]** | Maastricht Ageing Study,  Age 50-81  Netherlands | 949 | 51% | Dementia | 12 years | Alcohol consumption, coronary heart disease, physical activity, renal dysfunction, diabetes, high cholesterol, smoking, obesity, hypertension, Mediterranean diet, depression, high cognitive activity | AUC  0.75 (0.69, 0.80) |
|  |  |  |  | **Deckers et al 2020 [52]** | CAIDE study, Finland  Midlife:40-50y  Late-life 65-79 | 1,628 | 40% | Dementia | 30 years |  | **Harrell C**  Midlife: 0.67  Late-life: 0.53 |
|  |  |  |  | **Huque et al. 2023 [47]** | HRS ADAMS;  Age>70years  CHS-CS;  Age≥65  MAP,  Age >60 years,  USA. | HRS ADAMS (n = 856);  CHS-CS (n = 3370);  MAP (n = 2184) | HRS ADAMS: 47.5%  CHS-CS: 40.9%  MAP: 26.5% | Dementia | >5 Yrs | obesity, diabetes, depression, smoking, physical activity, cognitive activity, alcohol intake, hypertension, and coronary heart disease | **C statistic**  HRS-ADAMS: 0.53(0.47, 0.58)  CHS-CS cohort:  0.54 (0.51-0.57)  MAP:0.53 (0.48-0.57) |
| 39 | CogDrisk-AD tool | **Community sample** | **AD** | **Huque et al 2023 [47]** | HRS ADAMS;  Age>70years  CHS-CS;  Age≥65  MAP,  Age >60 years,  USA. | HRS ADAMS (n = 856);  CHS-CS (n = 3602);  MAP (n = 2184) | HRS ADAMS: 47.5%  CHS-CS: 40.9%  MAP: 26.5% | AD | >5 Yrs | Age, Gender, Education, midlife obesity, Diabetes, Depression, TBI, Smoking, Loneliness, Physical activity, Cognitive activity, Fish intake, stroke and midlife hypertension | **C statistic**  HRS-ADAMS: 0.67(0.59, 0.75)  CHS-CS cohort:  0.72 (0.69, 0.75)  MAP:0.65 (0.61-0.69) |
|  |  |  |  | **Kootar et al 2023 [49]** | SNACK-K,  Age: >60 years  Sweden | N=2137 | 36.6% | AD | 8 Years | Age, Gender, Education, Diabetes, Stroke, Hyperten­sion, Smoking, TBI, Depres­sion, Loneliness, Physical activity, Cognitive | **C statistic**  0.69 (0.60, 0.78) |
| Validation of tools that were developed using late life risk factors information | | | | | | | | | | | |
| 4 | Brief Dementia Screening Indicator (BDSI) | **Community**  >65-74 years | **Dementia** | **Stephan et al. 2020 [46]** | 10/66 Study  Age≥65 | 11,143 | 37% | Dementia  (10/66 diagnostic  algorithm) | Avg. 3.8 years | Age, sex, education, height-to-weight ratio, diabetes, depression total cholesterol, smoking, alcohol use, traumatic brain injury, physical activity, social engagement, and fish intake | **C statistic**  **0.68 (0.65-0.71)** |
| 21 | mCAIDE risk score | **Community sample** | Cognitive impairment | **Tolea et al 2021 [9]** | Clinical sample, USA  Age≥ 40 (mean age, 71y) | 449 | 40% | MCI or Demetia,  AD | N/A | Age, sex, education, BMI, SBP, high cholesterol, mini-PPT, | **C statistic**  MCI or Dementia: 0.78 (0.71-0.85)  AD: 0.82 (0.72-0.92) |
| 26 | Rapid Assessment of Dementia Risk (RADaR) | **Community sample** | Dementia | **Capuano et al 2022 [20]** | Religious Order Study (ROS);  Avg: 75.0±7 Minority Aging Research Study (MARS), USA  Avg: 73±6 | ROS: 1,299 MARS: 679 | ROS: 28% MARS: 22% | Dementia | ROS: 18 Yrs  MARS: 8 Years | Age, memory complaint, the ability to handle finances, the recall of the month, recall of the room, and recall of three words | **C-statistic (95% CI):**  **ROS:** 0.82 (0.78, 0.86)  **MARS:** 0.85 (0.80, 0.90) |
| 29 | Rotterdam Study Basic Dementia Risk Model (BDRM) | Community sample | Dementia | **Licher et al 2019 [21]** | EPOZ Study,  Mean 70.8 9sd 6.5, Netherlands  ADNI-1, Mean 75.9 (sd 4.9) USA | EPOZ: 514  ADNI-1: 228 | EPOZ: 46.7%  ADNI-1: 52% | Dementia | EPOZ: 9.5 years  ADNI-6.3 years | Age, history of symptomatic stroke, presence of subjective memory decline, need for assistance with finances or medication, cognitive testing, APOE-ε4 carrier status, brain MRI parameters | **C-statistic (95% CI):**  **ROS:**  0.75(0.67, 0.82)  **ADNI-1:**  0.72 (0.63, 0.83) |
|  |  |  |  | **Stephan et al 2020 [46]** | 10/66 Study  Age≥65 | 11,143 | 37% | Dementia,  (10/66 diagnostic  algorithm) | Avg. 3.8 years | Age, stroke, subjective memory decline, IADL | **C-statistic (95%CI):** 0.72 (0·69–0·74) |
| 31 | Simplifed Telephone Assessment for Dementia risk (STAD) | **Community Sample, Japan** | Dementia | **Makino 2021 [22]** | Community population, Japan  Age≥65 | 1750 | 54.2% | Dementia | 2 years | age, sex, education, hypertension,  heart disease, diabetes mellitus, hyperlipidaemia, drinking  habit, smoking habit, slow gait speed, living alone, physical  inactivity, and cognitive function at baseline | **AUC: 0.70** |
| 37 | Late life Dementia risk index | **Community sample. Age: 60-95** | Dementia | **Walters et al. 2016 [25]** | Routinely collected health data algorithm, THIN, UK  Age: 60-95 | 264224 | 48% | Dementia | 5 year | Age, sex, social deprivation, smoking, BMI, heavy alcohol use, anti-hypertensive drugs, diabetes, stroke/TIA, atrial fibrillation, aspirin, depression | **C statistic**  **Age: 65-80**  0.84 (0.81, 0.87)  Age: |
|  |  |  |  | **Anatürk et al., 2023 [12]** | UKB: 40-73 (Mean age: 60)  WHII: 35-55 (Mean age: 57) | UKB, n=44151,  WHII, n=2934 | UKB, 49.5%  Whitehall II study, 71.6% | Dementia | 14 years | Age, education, parental history of dementia, material deprivation, a history of diabetes, stroke, depression, hypertension, high cholesterol, household occupancy, and sex | **AUC (95% CI)**  **UKB:** 0.77 (0.76, 0.79)  **WHII:** 0.74 (0.69, 0.78) |
| 40 | DemNCD | **Community sample. Age: 60-95** | **Dementia** | **Huque et al., 2024[26]** | Atherosclerosis Risk in Communities  (ARIC);  CHS-CS; HRS ADAMS;  MAP,  FHS study, the  MRC  CFAS-I and CFAS-II), **Sydney-MAS**, the Maastricht Aging Study  (MAAS)  and  the Singapore Longitudinal Ageing Study-I (SLAS-I) .  Age≥65 | 14,613 | 41.6% | Dementia | Mean (SD): 7.1 (5.5) | Age, sex, education, obesity, smoking, alcohol consumption. high cholesterol, HDL, LDL, hypertension, diabetes, stroke, TBI, depression, physical activity, cognitive activity, loneliness, fish intake, fruits & vegetable intake, Atrial Fibrillation, sleep problem, hearing loss | **AUC:** 0.68 (0.67, 0.69) |
| 43 | Late-life Primary Care risk score | **Community sample. Age: ≥60** | **Dementia** | **Jung et al., 2024[6]** | Korean National Health Insurance Service database; Mean age: 66.7 | 616, 844 | 47% | Dementia | 6-years | Age, sex,  obesity, smoking, alcohol consumption, physical activity, diabetes, hypertension, dyslipidaemia, and  Chronic kidney disease. | **AUC:** 0.743 (0.742, 0.744) |
| Validation of tools that were developed using clinical risk factors information | | | | | | | | | | | |
| 1 | AgeCoDe | **Clinical,** Age ≥75 | **AD** | **Jessen 2011 [29]** | Clinical sample, Age≥75 | 1529 | 35% | AD  (NINCDS-ADRDA) | 18 months | Age, subjective memory impairment, verbal flunecy, delayed recall, MMSE, IADL | A0.79 (0.74–0.84) |
| 1 | AgeCode | **Clinical,** Age ≥75 | **AD** | **Stephan 2020 [46]** | 10/66 Study  Age≥65 | 11,143 | 37% | Dementia  (10/66 diagnostic  algorithm) | Avg. 3.8 years | Age, subjective memory impairment, verbal flunecy, delayed recall, MMSE, IADL | 0·66 (0·62–0·71) |
| 2 | AHEAD score | **Clinical,** Age 51-88 | **CVD**  Atrial Fibrillation | **Hu et al. 2019 [30]** | Registry data with heart failure, Taiwan, Mean age 72 | 387,595 | 52% | Dementia  (ICD-9-CM) | 2.91 Yrs | Atrial fibrillation, anaemia, haemoglobin level less than 130g/l for men and 120g/l for women elderly (age ≥ 70Yrs), abnormal renal parameters (creatinine ≥ 130μmol/l)and diabetes mellitus | 0.55 (0.54, 0.55) |
| 8 | CHA_2_DS­_2_ -VASC scores | **Clinical sample** | **CVD** | **Graves et al 2018 [38]** | Diagnosed with atrial fibrillation, USA | 74,081 | 54% | Dementia | 10 Years | Age, sex, Ischaemic stroke, Intracranial bleeding, Myocardial Infraction, CABG, Peripheral artery disease, hypertension, heart failure, diabetes, thyroid, thyrotoxicosis | **C statistic**  Male: 0.652 (0.537, 0.766)  Female: 0.730 (0.643, 0.817) |
|  |  |  |  | **Hu et al. 2019 [30]** | Diagnosed with heart failure, Taiwan | 387,595 | 52% | Dementia | Avg: 2.91 years | Age, sex, Ischaemic stroke, Intracranial bleeding, Myocardial Infraction, CABG, Peripheral artery disease, hypertension, heart failure, diabetes, thyroid, thyrotoxicosis | **C statistic**  0.61 (0.60, 0.61) |
| 13 | Dementia risk prediction model for Chinese type 2 diabetes patients | **Clinical sample** | Dementia | **Li et al 2018a [35]** | Diabetes registry,  Age: 50-94  Taiwan | 27,540 | 45% | Dementia | 10 years | Age, sex, duration of type 2 diabetes, BMI, variation in FPG, and HbA1c, stroke, hypoglycemia, postural hypotension, coronary artery disease and anti-diabetes medication | **C statistic**  0.75 (0.73, 0.77) |
| 14 | The dialysis dementia risk score (DSDRS) | **Clinical sample** | Dementia | **Exalto et al 2013 [36]** | Algorithmically classified Diabetes  based on records and in registry, USA | 29,961 | 53% | Dementia | 10 Years | Age, education, microvascular disease, diabetic foot, cerebrovascular disease, cardiovascular disease, acute metabolic event, depression | **C statistic**  0.74 |
| 16 | The Framingham risk score | **Clinical sample** | CVD | **Tolea et al 2021 [9]** | Clinical sample,  Age: 74.34±9.84, South Florida academic dementia center, USA | 219 | 50.2% | MCI or dementia | N/A | Age, sex, education, BMI, SBP, high cholesterol, mini-PPT, | **C statistic**  0.793 (0.643, 0.944) |
|  |  |  |  | **Li et al 2018b [53]** | Framingham Heart Study, USA  Age>60 years | 2,383 | 40% | Dementia | 30 years | Age, marital status, BMI, stroke, diabetes, ischemic attack, cancer | **C statistic**  0.716 |
| 17 | Intermountain Mortality Risk Score | **Clinical sample** | CVD | **Graves 2018 [38]** | Diagnosed with atrial fibrillation  Age: ≥18 | 74,081 | 54% | Dementia | 10 years | Age, sex, admission complete blood count and basic metabolic profile | **C statistic**  **Male:**0.68 (0.585-0.783)  **Female:** 0.69 (0.600, 0.79) |
| 23 | Mild Cognitive Impairment to Dementia Risk (CIDER) score | Clinical sample | Dementia | **Wang et al 2022a [39]** | PROMPT registries, CANADA  Age: 67 (sd 8)  ADNI, USA:  Age: 74 years,(SD 8.1). Diagnoses with MCI, USA and Canada | PROMPT: 452  ADNI: 598 | PROMPT: 57%  ADNI: 60% | Dementia | PROMPT: 3 years  ADNI: 17.4 years | Age, sex , education, Marital status, hypertension, Mood disorder, MOCA | **C-statistic (95% CI):** **PROMPT cohort:**  0.61 (0.59, 0.63) **ADNI cohort:** CIDER MoCA: 0.72 (0.69, 0.75) |
| 33 | The dialysis dementia risk score (DDRS) | **Clinical sample** | Dementia | **Ling 2022 [42]** | Patients with kidney failure, Taiwan  Age 40-80 | 6434 | 43.4% | Dementia | 12 years | Age, diabetes mellitus, stroke, anemia, hypertension, hyperlipidemia, depression, insomnia, TBI, Parkinson's disease, Myocardial Infraction, Atrial Fibrillation, Hyper/hypo-thyroids | **C statistic**  0.71 (0.69, 0.73) |
| 38 | Multipredictor risk models for AD | **Clinical sample** | AD | **Hou et al 2023 [43]** | ADNI, US and CANADA  Clinical sample | 195 | NA | AD | 5 years | Age, depression, diabetes, Systolic blood pressure, FAQ, RAVTL, LM-DR, ADAS11 | **AUC:** 0.75 (0.63, 0.85) |
| 45 | FDRS | **Clinical sample** | AD | **Pan 2024**[28] | ROSMAP study | 500 | NA | AD | NA | ge, geriatric depression, diastolic blood pressure, Systolic blood pressure, Heart rate, APOE, Neurological disorder, Present living arrangements, Primary occupation, difficulty with memory, Marital status | **AUC: 0.82 (0.77-0.86)** |

**Table S3. AD Risk factors considered in developing AD risk scores.**

| **Risk factors** | | **Community settings from cohort studies** | **Clinical settings** |
| --- | --- | --- | --- |
| **Genetics** | **APOE ε4 status** | BDRM; LOAD Risk Score; Late-life dementia risk index, | FDRS |
| **Demographics** | **Age** | ANU-ADRI; CogDrisk-AD; LOAD Risk Score; Late-life dementia risk index; ZARADEMP AD Risk Score;  FDRS-DMSV; | AgeCoDe; Multipredictor risk models for AD; FDRS; |
|  | **Sex** | ANU-ADRI; CogDrisk-AD; LOAD Risk Score; ZARADEMP AD Risk Score;  FDRS-DMSV; | Clinical index for aMCI to probAD conversion; |
|  | **Ethnicity** | LOAD Risk Score; |  |
|  | **Marital status** | ZARADEMP AD Risk Score;  FDRS-DMSV; | FDRS; |
|  | **Education** | ANU-ADRI; CogDrisk-AD; LOAD Risk Score; ZARADEMP AD Risk Score; |  |
| **Lifestyles** | **Health Check** |  | Multipredictor risk models for AD; |
|  | **BMI** | ANU-ADRI; CogDrisk-AD; LOAD Risk Score; Late-life dementia risk index; ZARADEMP AD Risk Score; |  |
|  | **Smoking** | ANU-ADRI; CogDrisk-AD; LOAD Risk Score; |  |
|  | **Alcohol** | ANU-ADRI; Late-life dementia risk index; |  |
|  | **Healthy diet** | ANU-ADRI; |  |
|  | **Physical activity** | ANU-ADRI, CogDrisk-AD; |  |
|  | **High Cholesterol** | ANU-ADRI: CogDrisk-AD; LOAD Risk Score |  |
|  | **Diabetes** | ANU-ADRI; CogDrisk-AD;LOAD Risk Score; | Multipredictor risk models for AD; |
|  | **Ventricular hypertrophy** | Late-life dementia risk index; |  |
|  | **Hypertension/SBP** | LOAD Risk Score; CogDrisk-AD; FDRS-DMSV; | Multipredictor risk models for AD; FDRS; |
|  | **Head Injury** | ANU-ADRI; CogDrisk-AD |  |
|  | **Depression/Mood** | ANU-ADRI; CogDrisk-AD; ZARADEMP AD Risk Score;  FDRS-DMSV; | Multipredictor risk models for AD; FDRS; |
|  | **Hearing loss** | ZARADEMP AD Risk Score; |  |
|  | **Hyperlipemia/dyslipidemia** | LOAD Risk Score; |  |
|  | **Stroke** | BDRM; CogDrisk-AD |  |
|  | **Cerebrovascular disease** | BDRM |  |
| **Cognition** | **Social engagement** | ANU-ADRI; CogDrisk-AD |  |
|  | **cognitive engagement** | ANU-ADRI; CogDrisk-AD |  |
|  | **Cognitive function** | BDRM; Late-life dementia risk index; FDRS-DMSV; | AgeCoDe; Clinical index for aMCI to probAD conversion; Risk tool for aMCI to probAD; FDRS; |
|  | **Functional dependence** | BDRM; Late-life dementia risk index | AgeCoDe; Clinical index for aMCI to probAD conversion; Risk tool for aMCI to probAD; Multipredictor risk models for AD; |
| **Environment** | **Pesticide exposure** | ANU-ADRI; CogDrisk-AD |  |
| **Abnormal brain  parameters** | **Brain MRI** | BDRM; Late-life dementia risk index; FDRS-DMSV; | Risk tool for aMCI to probAD; |

**Table S4. Prediction model Risk of Bias Assessment for all the studies considered in this review.**

| Study ID | Author, Year | Risk of Bias (ROB) | | | | Applicability | | | Overall | |
| --- | --- | --- | --- | --- | --- | --- | --- | --- | --- | --- |
|  |  | Participants | Predictors | Outcome | Analysis | Participants | Predictors | Outcome | ROB | Applicability |
| 1 | Huque et al. 2024 | + | - | + | + | + | + | + | - | + |
| 2 | Anaturk et al. 2023 | + | + | - | - | + | - | + | - | - |
| 3 | Anstey et al. 2014 | + | + | + | - | + | + | + | - | + |
| 4 | Barnes et al 2009 | + | + | - | - | + | - | + | - | - |
| 5 | Barnes et al 2014a | + | + | + | - | + | + | + | - | + |
| 6 | Barnes et al 2014b | + | - | - | - | + | - | + | - | - |
| 7 | Capuano et al., 2022 | + | - | + | - | + | + | + | - | + |
| 8 | Chosy et al., 2019 | + | + | - | + | - | + | - | - | - |
| 9 | Deckers et al.,2020 | + | + | - | - | + | + | + | - | + |
| 10 | Dharmasaroja et al 2022 | + | + | + | - | - | + | + | - | - |
| 11 | Downer et al 2016 | + | + | - | + | - | + | + | - | - |
| 12 | Exalto et al 2013 | + | + | + | - | + | + | + | - | + |
| 13 | Exalto et al 2014 | + | + | + | - | + | + | + | - | + |
| 14 | Graves et al 2018 |  | + | + | - | - | + | - | - | - |
| 15 | Hou et al 2023 | + | - | + | + | - | - | - | - | - |
| 16 | Hu et al 2019 | + | - | + | - | + | + | + | - | + |
| 17 | Ibarrondo et al 2022 | + | + | + | + | + | + | + | + | + |
| 18 | Jessen et al 2011 | + | - | - | - | + | - | + | - | - |
| 19 | Kivipelto et al 2006 | + | + | + | - | + | + | + | - | + |
| 20 | Lee at al 2014 | + | - | + | - | - | + | + | - | - |
| 21 | Li et al 2018a | - | - | - | - | + | + | + | - | + |
| 22 | Li et al 2018b | + | + | + | - | + | + | + | - | + |
| 23 | Licher et al 2019 | + | + | + | + | - | + | - | + | - |
| 24 | Lin et al 2018 | - | + | - | + | - | + | - | - | - |
| 25 | Ling et al 2022 | + | + | - | - | - | + | + | - | - |
| 26 | Makino et al 2021 | + | + | + | - | + | + | + | - | + |
| 27 | Mehta et al 2016 | - |  |  | + | + | - | + |  | - |
| 28 | Ng et al 2021 | + | + | + | - | + | + | - | - | - |
| 29 | Rawtaer et al 2016 | + | + | + | - | + | + | - | - | - |
| 30 | Ren et al 2022 | + | + | - | + | + | + | + | - | + |
| 31 | Schiepers et al 2018 | + | + | + | - | + | + | + | - | + |
| 32 | Stephan et al 2020 | + | + | + | - | + | + | + | - | + |
| 33 | Tolea et al 2021 | - | - | - | - | - | - | - | - | - |
| 34 | Vuoksimaa et al 2016 | + | + | - | - | + | + | - | - | - |
| 35 | Wang et al 2022a | + | + | - | - | + | + | + | - | + |
| 36 | Wang et al 2022b | - | + | + | - | + | + | + | - | - |
| 37 | Huque et al 2023 | + | + | + | + | + | + | + | + | + |
| 38 | Kootar et al 2023 | + | + | + | + | + | + | + | + | + |
| 39 | Walters et al 2016 | + | + | + | + | + | + | + | + | + |
| 40 | Vos et al 2017 | + | - | - | - | + | + | - | - | - |
| 41 | Chen et al 2024 | + | + | + | - | + | - | + | - | - |
| 42 | Jung et al 2024 | + |  | + | - | + | - | + | - | - |
| 43 | Pan et al 2024 | - | + |  | - | - | - |  | - | - |
| 44 | Santabárbara et al 2021 | + | + | + | - | + | + | + | - | + |
| 45 | Anstey et al 2013 |  | + | + | + | + | + | + |  | + |
| 46 | Eskelinen et al 2011 | - | - | + | - | - | + | + | - | - |
| 47 | Liang et al 2020 | + | + | + | - | + | + | + | - | + |
| 48 | Anstey et al 2022 |  | + | + | + | + | + | + |  | + |
| 49 | Reitz et al 2010 | - | + | + | - | + | - | + | - | - |

Note: ‘+’ indicates low risk of bias, ‘-‘high risk of bias and empty cell ‘ ‘ indicates uncertain risk of bias.

**Table S5 Estimated relative costs and health services required for administering each Dementia Risk Tool in a clinical or community setting.**

| **Tool Category** | **ID** |  | **Data sources for Risk Score** | **Cost ($USD)** |
| --- | --- | --- | --- | --- |
| Midlife | 5 | CAIDE midlife healthy-diet index | Patient time(10min) |  |
|  | 6 | CAIDE risk score | GP HA(<30min) + Pathology + Genotyping | $150.76 |
|  | 7 | Cardiovascular health (CVH) metrics | GP HA(<30min) + Pathology | $53.74 |
|  | 15 | Education & Occupation midlife Risk | GP HA(<30min) | $42.59 |
|  | 27 | Risk score for late risk of dementia | GP HA(<60min) + Dietician Assessment | $143.67 |
|  | 42 | Midlife primary care risk tool | GP HA(<60min) + Pathology | $110.12 |
| Midlife to Late-life | 3 | ANU-ADRI risk score | Patient time(20min) |  |
|  | 9 | Clinical Dementia Risk Score | Patient time(20min) |  |
|  | 12 | CogDrisk Tool | Patient time(20min) |  |
|  | 20 | LIBRA risk score | Patient time(20min) |  |
|  | 21 | mCAIDE risk score | GP HA(<60min) | $98.97 |
|  | 24 | Modified dementia risk scores(MDRS) | GP HA(<30min) + Pathology + Genotyping | $150.76 |
|  | 25 | Neurocognitive Disorders Risk Score | GP HA(<30min) | $42.59 |
|  | 32 | SLAS Risk Index | Patient time(10min) |  |
|  | 36 | UKBDRS | GP HA(<60min) + Pathology + Genotyping | $207.14 |
|  | 39 | CogDrisk-AD Tool | Patient time(20min) |  |
| Late-Life | 4 | Brief Dementia Screening Indicator (BDSI) | GP HA(<30min) | $42.59 |
|  | 18 | JAGES risk index | GP HA(<30min) + Pathology + Dietician Ax | $154.82 |
|  | 19 | LOAD Risk Score | GP HA(<60min) + Pathology + Genotyping | $207.14 |
|  | 22 | MADeN Risk score | GP HA(<60min) | $98.97 |
|  | 26 | RADaR | GP HA(<60min) | $98.97 |
|  | 29 | BDRM Risk score | GP HA(<60min) + MRI + Genotyping | $474.11 |
|  | 31 | STAD Risk score | GP HA(<30min) | $42.59 |
|  | 34 | The late-life dementia risk index | GP HA(<60min) + MRI + Carotid Ultrasound + Genotyping | $593.87 |
|  | 35 | ZARADEMP AD Risk Score | GP HA(<30min) | $42.59 |
|  | 37 | Routine Primary Care data model | GP HA(<60min) + Pathology | $110.12 |
|  | 40 | DemNCDrisk | Patient time(20min) |  |
|  | 44 | Florey Dementia Risk Score (FDRS-DMSV) | GP HA(<60min) | $98.97 |
|  | 41 | Polysocial risk score | Patient time(20min) + Genotyping (GWAS) | $497.70 |
|  | 43 | Late-life primary care risk score | GP HA(<30min) + Blood Pathology Test | $110.12 |
| Clinical | 1 | AgeCoDe Model | GP HA(60min) | $192.94 |
|  | 2 | AHEAD scores | GP HA(<30min) + Pathology + Haematology | $64.42 |
|  | 8 | CHA2DS2-VASc scores | GP HA(<30min) + Pathology + Haematology | $53.27 |
|  | 10 | Index to predict aMCI to AD conversion | Memory Clinic | $399.80 |
|  | 11 | HASTE Risk score | GP HA(<30min) + MRI | $320.70 |
|  | 13 | Dementia risk for Chinese T2DM patients | GP HA(<60min) + Pathology | $110.12 |
|  | 14 | DSDRS | GP HA(<60min) | $98.97 |
|  | 16 | Framingham risk score | GP HA(<30min) | $42.59 |
|  | 17 | Intermountain Mortality Risk Score | GP HA(<30min) + Pathology + Haematology | $64.42 |
|  | 23 | CIDER Score | GP HA(<60min) | $98.97 |
|  | 28 | Risk tool to predict conversion aMCI to AD | Memory Clinic + MRI | $677.91 |
|  | 30 | RxDxDementia Risk Index | GP HA(<60min) | $98.97 |
|  | 33 | The dialysis dementia risk score (DDRS) | GP HA(<60min) | $98.97 |
|  | 38 | Multipredictor risk score (ADNI) | GP HA(<60min) + MRI + Neuropsych. Ax + CSF + Genotyping | $939.08 |
|  | 45 | Florey Dementia Risk Score (FDRS) | GP HA(<60min) + Genotyping | $195.99 |

Note: *$USD0.63=$AUD1; Costings are estimates only for the purpose of comparison across tools.

Cost estimates based on Australian MBS fees as at Februfor GP time for health assessment (GP HA) (Items 701,703,705,707); Memory Clinic (Item 145); Dietician (Item 10954), Brain MRI (63001); Carotid Ultrasound (55274); Pathology and Haematology (66512, 65070); APOE4 genotyping and GWAS analysis (commercial service estimates); CSF biomarkers (39000, and commercial services estimate).


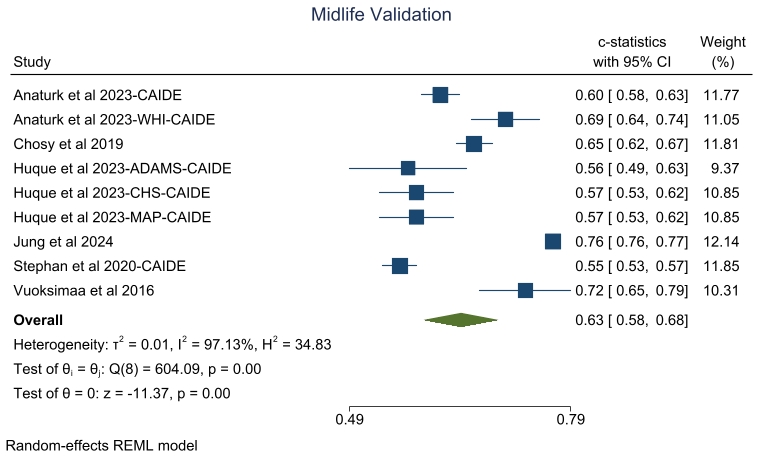


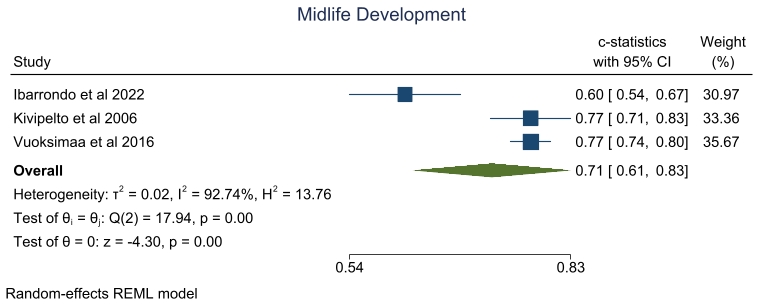


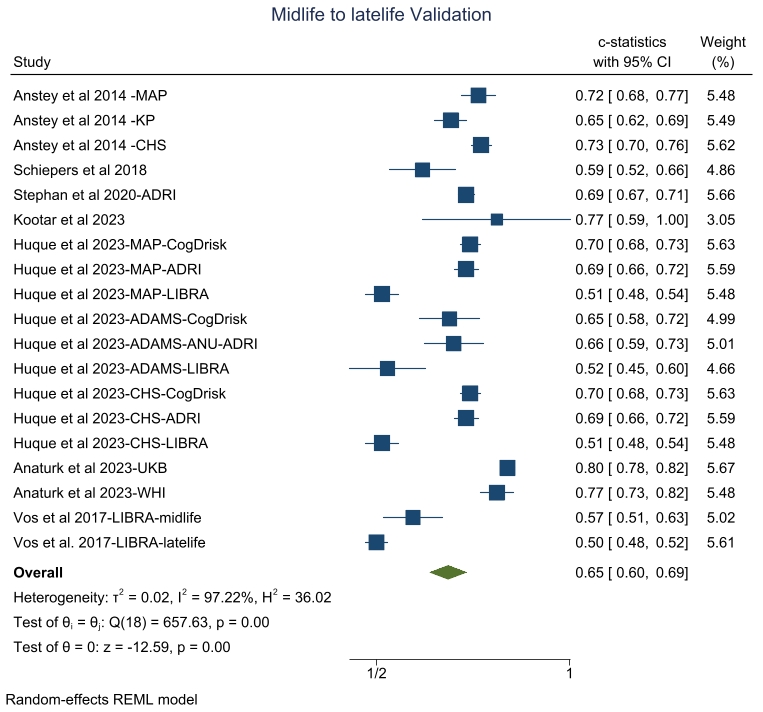


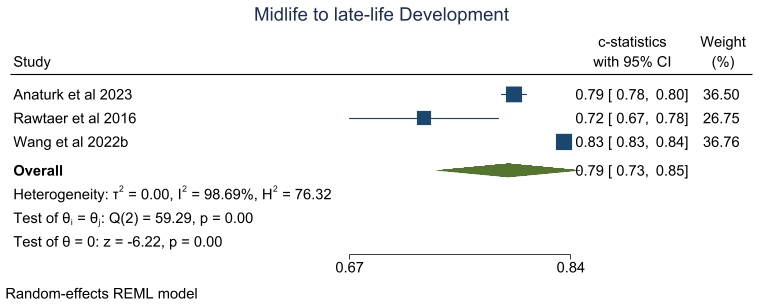


**Figure S1: Pooled c-statistics of the developed and validation of midlife (left panel) and midlife to late-life dementia risk assessment tools (right panel)**


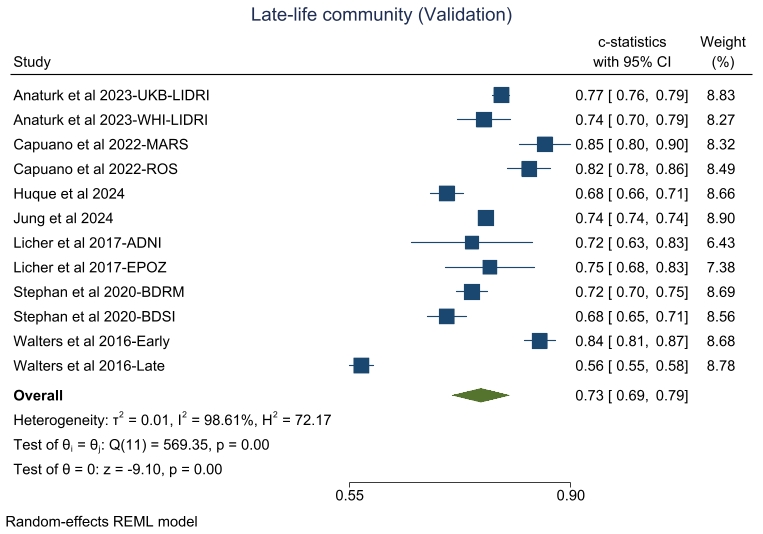


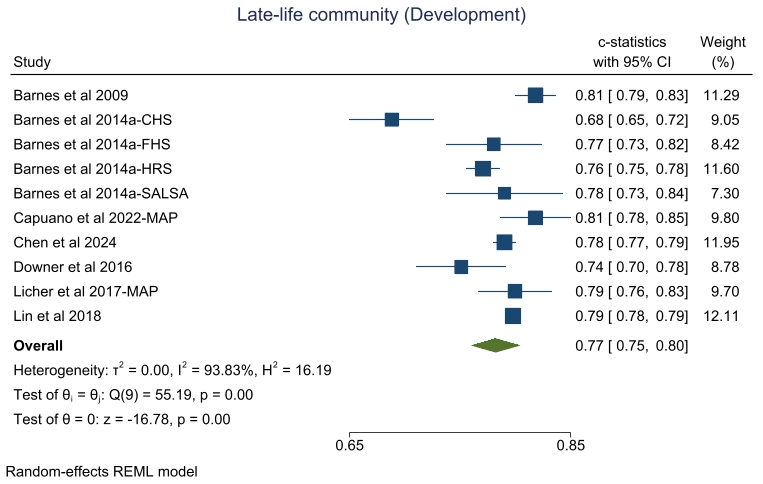


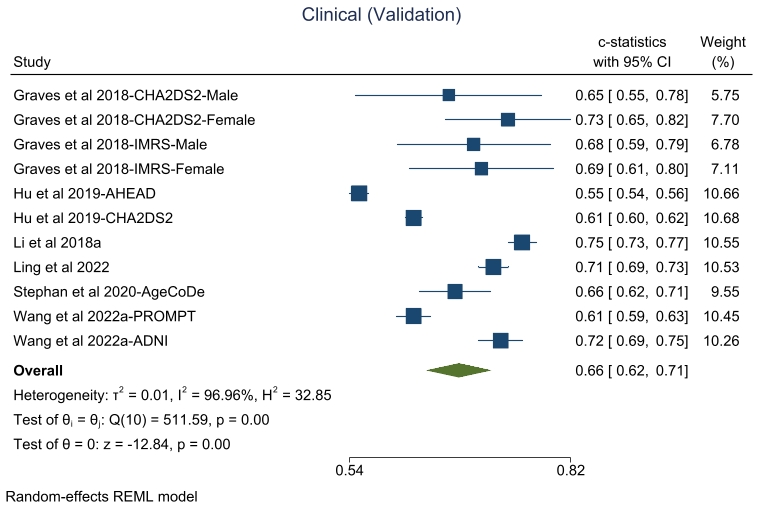


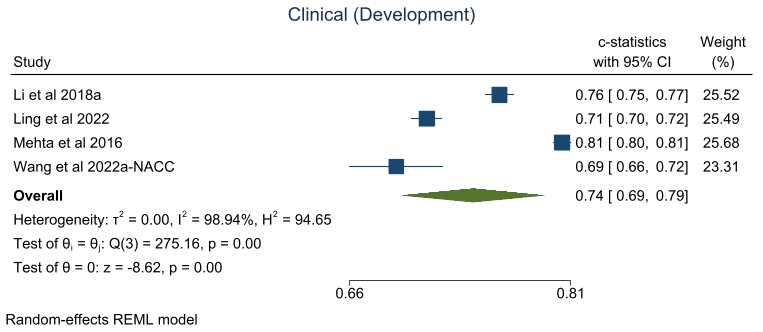


**Figure S2: Pooled c-statistics of the developed and validation of community-based late-life (left panel) and clinical dementia risk assessment tools (right panel)**


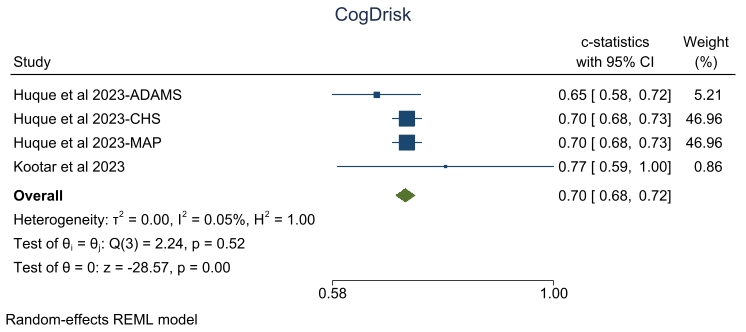


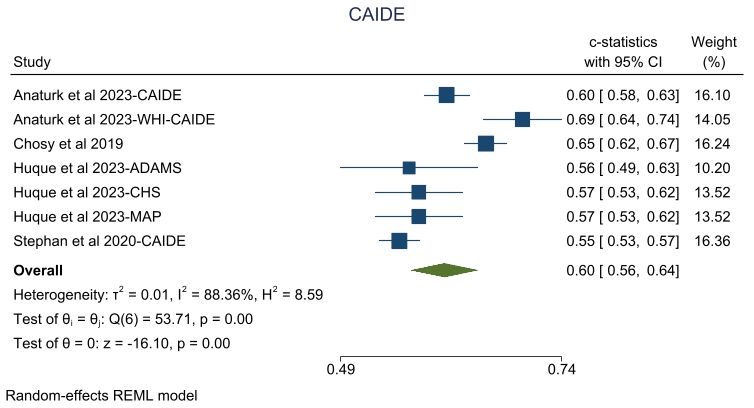


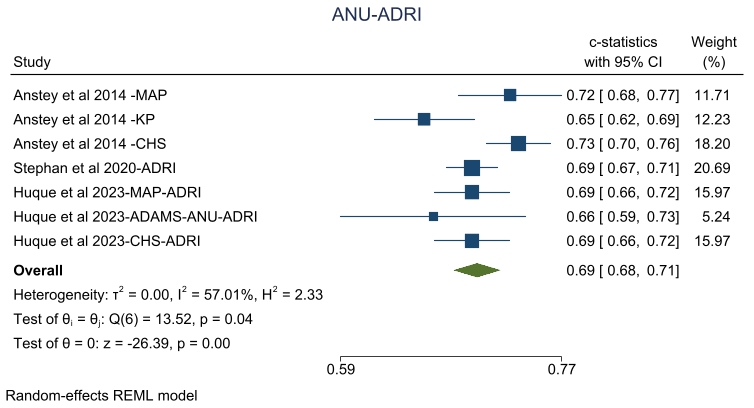


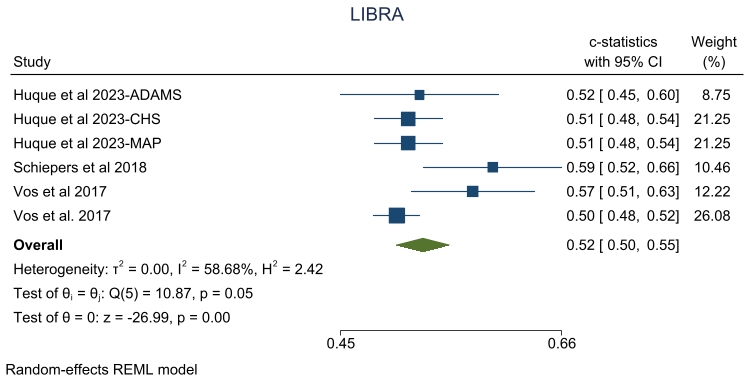


**Figure S3: Pooled c-statistics of the widely validated dementia risk assessment tools.**


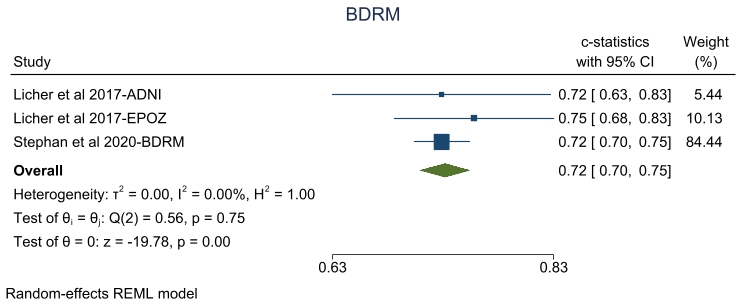


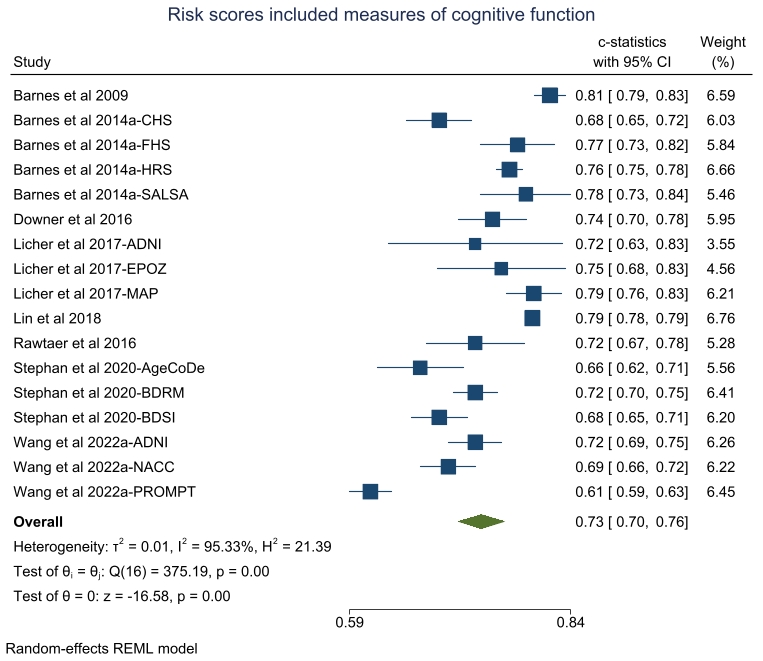


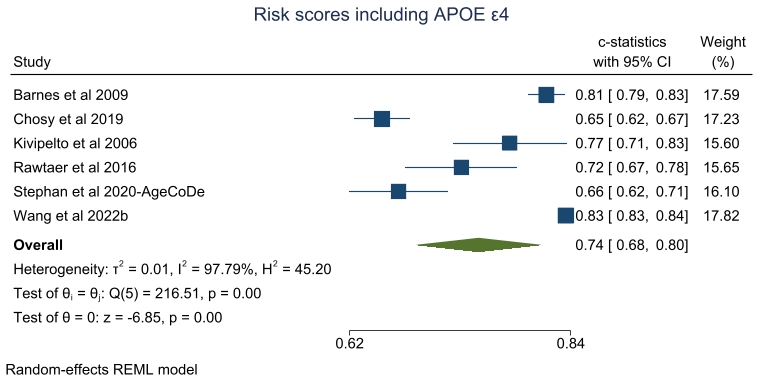


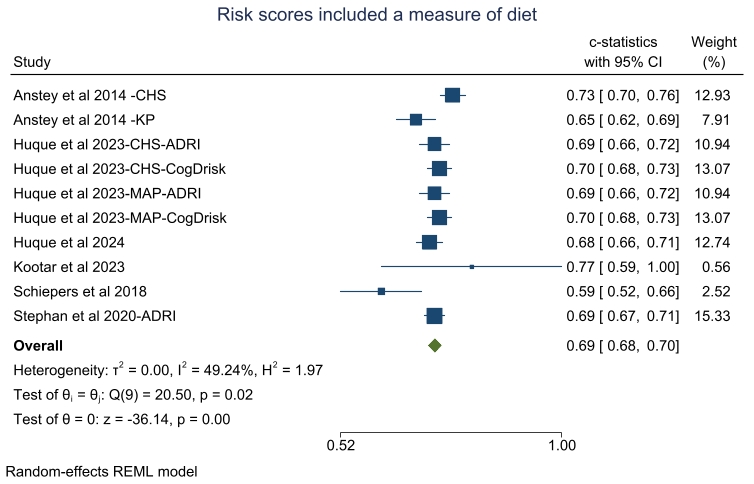


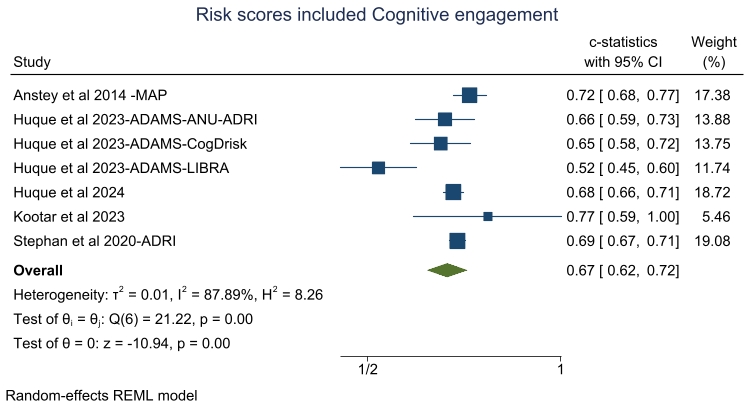


**Figure S4: c-statistics dementia risk scores with various predictors**


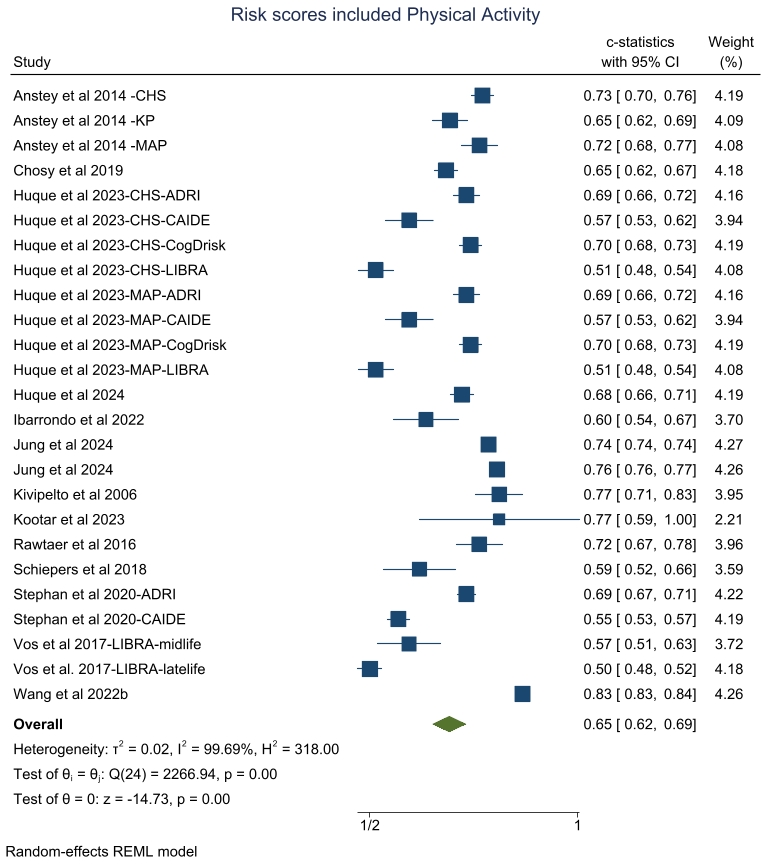


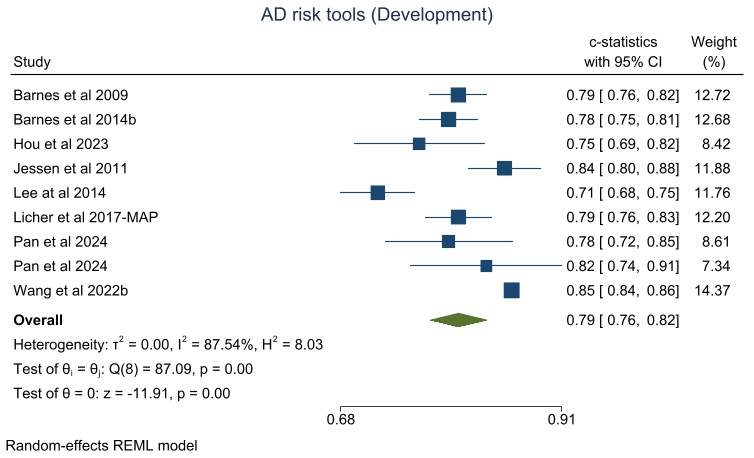


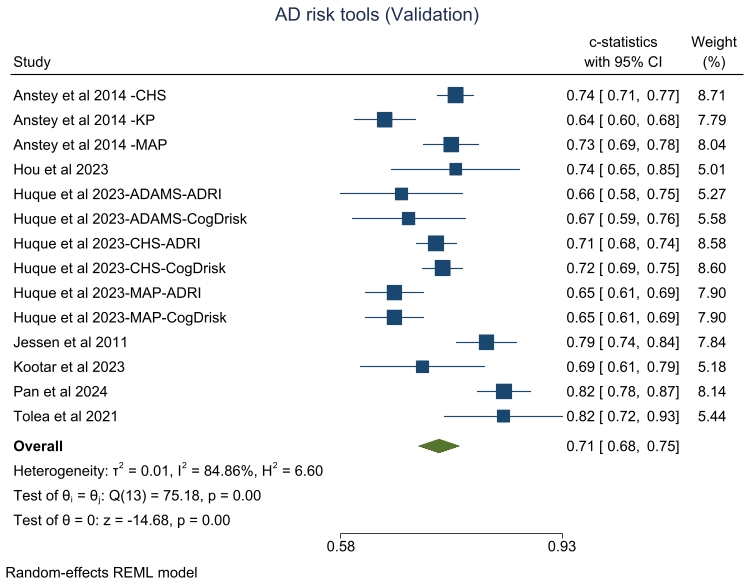


**Figure S5 Pooled c-statistics of the development and validation of AD risk assessment tools.**


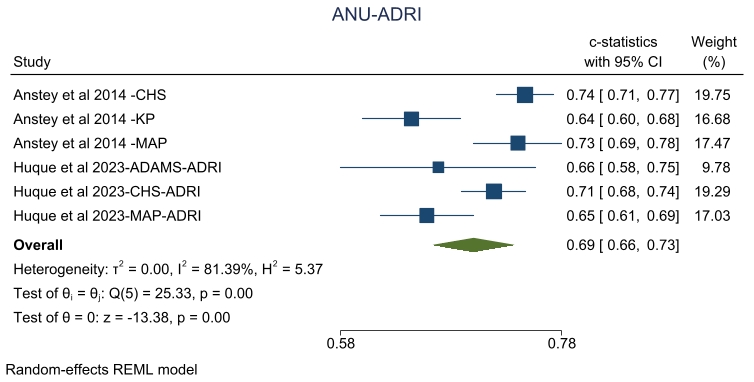


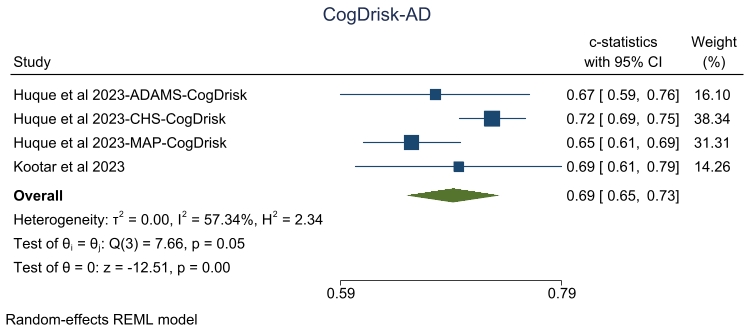


**Figure S6 Pooled c-statistics of the widely validated AD risk assessment tools.**

**References: Studies selected for tools identification and quantitative synthesis.**

1. M.H. Eskelinen, T. Ngandu, J. Tuomilehto, H. Soininen, and M. Kivipelto, *Midlife healthy-diet index and late-life dementia and Alzheimer's disease* Dement Geriatr Cogn Dis Extra, 2011. **1**(1): p. 103-12.

2. M. Kivipelto, T. Ngandu, T. Laatikainen, B. Winblad, H. Soininen, and J. Tuomilehto, *Risk score for the prediction of dementia risk in 20 years among middle aged people: a longitudinal, population-based study* Lancet Neurol, 2006. **5**(9): p. 735-41.

3. O. Ibarrondo, J.M. Huerta, P. Amiano, M.E. Andreu-Reinon, O. Mokoroa, E. Ardanaz, R. Larumbe, S.M. Colorado-Yohar, F. Navarro-Mateu, M.D. Chirlaque, and J. Mar, *Dementia Risk Score for a Population in Southern Europe Calculated Using Competing Risk Models* J Alzheimers Dis, 2022. **86**(4): p. 1751-1762.

4. Y. Liang, T. Ngandu, T. Laatikainen, H. Soininen, J. Tuomilehto, M. Kivipelto, and C. Qiu, *Cardiovascular health metrics from mid- to late-life and risk of dementia: A population-based cohort study in Finland* PLoS Med, 2020. **17**(12): p. e1003474.

5. E. Vuoksimaa, J.O. Rinne, N. Lindgren, K. Heikkila, M. Koskenvuo, and J. Kaprio, *Middle age self-report risk score predicts cognitive functioning and dementia in 20-40 years* Alzheimers Dement (Amst), 2016. **4**: p. 118-125.

6. W. Jung, S.H. Park, S. Kim, J. Lee, J. Park, S.-M. Jeong, S.-Y. Lee, K. Han, and D.W. Shin, *Predicting mid-and late-life dementia risk in primary care: A prognostic study from a national health screening cohort* Psychiatry Research, 2024. **342**: p. 116237.

7. Z.T. Wang, Y. Fu, Y.R. Zhang, S.D. Chen, S.Y. Huang, L. Yang, H.Q. Li, Y.N. Ou, J.F. Feng, Q. Dong, W. Cheng, L. Tan, H.F. Wang, and J.T. Yu, *Modified dementia risk score as a tool for the prediction of dementia: a prospective cohort study of 239745 participants* Transl Psychiatry, 2022. **12**(1): p. 509.

8. I. Rawtaer, L. Feng, V.H. Yuen, J. Li, M.S. Chong, W.S. Lim, T.S. Lee, C. Qiu, L. Feng, E.H. Kua, and T.P. Ng, *A Risk Score for the Prediction of Neurocognitive Disorders among Community-Dwelling Chinese Older Adults* Dement Geriatr Cogn Disord, 2016. **41**(5-6): p. 348-58.

9. M.I. Tolea, J. Heo, S. Chrisphonte, and J.E. Galvin, *A Modified CAIDE Risk Score as a Screening Tool for Cognitive Impairment in Older Adults* J Alzheimers Dis, 2021. **82**(4): p. 1755-1768.

10. L. Ren, J. Liang, F. Wan, Y. Wang, and X.J. Dai, *Development of a Clinical Risk Score Prediction Tool for 5-, 9-, and 13-Year Risk of Dementia* JAMA Netw Open, 2022. **5**(11): p. e2242596.

11. T.P. Ng, T.S. Lee, W.S. Lim, M.S. Chong, P. Yap, C.Y. Cheong, K.B. Yap, I. Rawtaer, T.M. Liew, Q. Gao, X. Gwee, M.P.E. Ng, S.O. Nicholas, and S.L. Wee, *Development, Validation and Field Evaluation of the Singapore Longitudinal Ageing Study (SLAS) Risk Index for Prediction of Mild Cognitive Impairment and Dementia* J Prev Alzheimers Dis, 2021. **8**(3): p. 335-344.

12. M. Anatürk, R. Patel, K.P. Ebmeier, G. Georgiopoulos, D. Newby, A. Topiwala, A.-M.G. de Lange, J.H. Cole, M.G. Jansen, A. Singh-Manoux, M. Kivimäki, and S. Suri, *Development and validation of a dementia risk score in the UK Biobank and Whitehall II cohorts* BMJ Mental Health, 2023. **26**(1): p. e300719.

13. K.J. Anstey, N. Cherbuin, and P.M. Herath, *Development of a new method for assessing global risk of Alzheimer's disease for use in population health approaches to prevention* Prev Sci, 2013. **14**(4): p. 411-21.

14. K. Deckers, M.P. van Boxtel, O.J. Schiepers, M. de Vugt, J.L. Muñoz Sánchez, K.J. Anstey, C. Brayne, J.F. Dartigues, K. Engedal, and M. Kivipelto, *Target risk factors for dementia prevention: a systematic review and Delphi consensus study on the evidence from observational studies* International journal of geriatric psychiatry, 2015. **30**(3): p. 234-246.

15. K.J. Anstey, S. Kootar, M.H. Huque, R. Eramudugolla, and R. Peters, *Development of the CogDrisk tool to assess risk factors for dementia* Alzheimers Dement (Amst), 2022. **14**(1): p. e12336.

16. D.E. Barnes, A.S. Beiser, A. Lee, K.M. Langa, A. Koyama, S.R. Preis, J. Neuhaus, R.J. McCammon, K. Yaffe, S. Seshadri, M.N. Haan, and D.R. Weir, *Development and validation of a brief dementia screening indicator for primary care* Alzheimers Dement, 2014. **10**(6): p. 656-665.e1.

17. H.R. Lin, T. Tsuji, K. Kondo, and Y. Imanaka, *Development of a risk score for the prediction of incident dementia in older adults using a frailty index and health checkup data: The JAGES longitudinal study* Prev Med, 2018. **112**: p. 88-96.

18. C. Reitz, M.X. Tang, N. Schupf, J.J. Manly, R. Mayeux, and J.A. Luchsinger, *A summary risk score for the prediction of Alzheimer disease in elderly persons* Arch Neurol, 2010. **67**(7): p. 835-41.

19. B. Downer, A. Kumar, S.P. Veeranki, H.B. Mehta, M. Raji, and K.S. Markides, *Mexican-American Dementia Nomogram: Development of a Dementia Risk Index for Mexican-American Older Adults* J Am Geriatr Soc, 2016. **64**(12): p. e265-e269.

20. A.W. Capuano, R.C. Shah, P. Blanche, R.S. Wilson, L.L. Barnes, D.A. Bennett, and Z. Arvanitakis, *Derivation and validation of the Rapid Assessment of Dementia Risk (RADaR) for older adults* PLoS One, 2022. **17**(3): p. e0265379.

21. S. Licher, M.J.G. Leening, P. Yilmaz, F.J. Wolters, J. Heeringa, P.J.E. Bindels, I. Alzheimer's Disease Neuroimaging, M.W. Vernooij, B.C.M. Stephan, E.W. Steyerberg, M.K. Ikram, and M.A. Ikram, *Development and Validation of a Dementia Risk Prediction Model in the General Population: An Analysis of Three Longitudinal Studies* Am J Psychiatry, 2019. **176**(7): p. 543-551.

22. K. Makino, S. Lee, S. Bae, I. Chiba, K. Harada, O. Katayama, Y. Shinkai, and H. Shimada, *Development and validation of new screening tool for predicting dementia risk in community-dwelling older Japanese adults* J Transl Med, 2021. **19**(1): p. 448.

23. D.E. Barnes, K.E. Covinsky, R.A. Whitmer, L.H. Kuller, O.L. Lopez, and K. Yaffe, *Predicting risk of dementia in older adults: The late-life dementia risk index* Neurology, 2009. **73**(3): p. 173-9.

24. J. Santabarbara, J. Bueno-Notivol, D.M. Lipnicki, C. de la Camara, R. Lopez-Anton, A. Lobo, and P. Gracia-Garcia, *A Novel Score for Predicting Alzheimer's Disease Risk from Late Life Psychopathological and Health Risk Factors* Int J Environ Res Public Health, 2021. **18**(4): p. 1-14.

25. K. Walters, S. Hardoon, I. Petersen, S. Iliffe, R.Z. Omar, I. Nazareth, and G. Rait, *Predicting dementia risk in primary care: development and validation of the Dementia Risk Score using routinely collected data* BMC Medicine, 2016. **14**(1): p. 6.

26. M.H. Huque, S. Kootar, K.M. Kiely, C.S. Anderson, M. van Boxtel, H. Brodaty, P.S. Sachdev, M. Carlson, A.L. Fitzpatrick, R.A. Whitmer, M. Kivipelto, L. Jorm, S. Köhler, N.T. Lautenschlager, O.L. Lopez, J.E. Shaw, F.E. Matthews, R. Peters, and K.J. Anstey, *A single risk assessment for the most common diseases of ageing, developed and validated on 10 cohort studies* BMC Medicine, 2024. **22**(1): p. 501.

27. S. Chen, S. Chen, K. Hanewald, Y. Si, H. Bateman, B. Li, X. Xu, S. Samtani, C. Wu, and H. Brodaty, *Social environment, lifestyle, and genetic predisposition with dementia risk: A long-term longitudinal study among older adults* The Journals of Gerontology, Series A: Biological Sciences and Medical Sciences, 2024. **79**(7): p. glae128.

28. Y. Pan, C. Chu, Y. Wang, Y. Wang, G. Ji, C.L. Masters, B. Goudey, and L. Jin, *Development and validation of the Florey Dementia Risk Score web-based tool to screen for Alzheimer's disease in primary care* EClinicalMedicine, 2024. **76**.

29. F. Jessen, B. Wiese, H. Bickel, S. Eiffländer-Gorfer, A. Fuchs, H. Kaduszkiewicz, M. Köhler, T. Luck, E. Mösch, M. Pentzek, S.G. Riedel-Heller, M. Wagner, S. Weyerer, W. Maier, and H. van den Bussche, *Prediction of dementia in primary care patients* PLoS One, 2011. **6**(2): p. e16852.

30. W.S. Hu and C.L. Lin, *Comparison of CHA(2)DS(2)-VASc and AHEAD scores for the prediction of incident dementia in patients hospitalized for heart failure: a nationwide cohort study* Intern Emerg Med, 2019. **14**(3): p. 395-402.

31. J. Spinar, J. Jarkovsky, L. Spinarova, A. Mebazaa, E. Gayat, J. Vitovec, A. Linhart, P. Widimsky, R. Miklik, and K. Zeman, *AHEAD score—Long-term risk classification in acute heart failure* International journal of cardiology, 2016. **202**: p. 21-26.

32. A.J. Camm, G. Lip, R. De Caterina, I. Savelieva, D. Atar, S.H. Hohnloser, G. Hindricks, and P. Kirchhof, *2012 focused update of the ESC Guidelines for the management of atrial fibrillation* an update of the 2010 ESC Guidelines for the management of atrial fibrillation--developed with the special contribution of the European Heart Rhythm Association, 2013. **14**: p. 1385-1413.

33. S. Lee, C. Ritchie, K. Yaffe, I. Cenzer, and D. Barnes, *A clinical index to predict progression from mild cognitive impairment to dementia due to Alzheimer's disease* PLoS ONE 9(12) (no pagination), 2014 Article Number: e113535 Date of Publication: 08 Dec 2014, 2014.

34. P.A. Dharmasaroja and T. Charernboon, *Clinical Risk Score for Predicting Vascular Dementia after Ischemic Stroke in Thailand* Stroke Res Treat, 2022. **2022**: p. 1600444.

35. C.I. Li, T.C. Li, C.S. Liu, L.N. Liao, W.Y. Lin, C.H. Lin, S.Y. Yang, J.H. Chiang, and C.C. Lin, *Risk score prediction model for dementia in patients with type 2 diabetes* Eur J Neurol, 2018. **25**(7): p. 976-983.

36. L.G. Exalto, G.J. Biessels, A.J. Karter, E.S. Huang, W.J. Katon, J.R. Minkoff, and R.A. Whitmer, *Risk score for prediction of 10 year dementia risk in individuals with type 2 diabetes: a cohort study* Lancet Diabetes Endocrinol, 2013. **1**(3): p. 183-90.

37. P.W. Wilson, R.B. D’Agostino, D. Levy, A.M. Belanger, H. Silbershatz, and W.B. Kannel, *Prediction of coronary heart disease using risk factor categories* Circulation, 1998. **97**(18): p. 1837-1847.

38. K.G. Graves, H.T. May, V. Jacobs, K.U. Knowlton, J.B. Muhlestein, D.L. Lappe, J.L. Anderson, B.D. Horne, and T.J. Bunch, *CHA(2)DS(2)-VASc scores and Intermountain Mortality Risk Scores for the joint risk stratification of dementia among patients with atrial fibrillation* Heart Rhythm, 2019. **16**(1): p. 3-9.

39. M. Wang, T.T. Sajobi, Z. Ismail, D. Seitz, T. Chekouo, N.D. Forkert, K. Fischer, A. Mackie, D. Pearson, D. Patry, A. Cieslak, B. Menon, P. Barber, B. McLane, R. Granger, D.B. Hogan, E.E. Smith, and I. Alzheimer's Disease Neuroimaging, *A pragmatic dementia risk score for patients with mild cognitive impairment in a memory clinic population: Development and validation of a dementia risk score using routinely collected data* Alzheimers Dement (N Y), 2022. **8**(1): p. e12301.

40. D.E. Barnes, I.S. Cenzer, K. Yaffe, C.S. Ritchie, S.J. Lee, and I. Alzheimer's Disease Neuroimaging, *A point-based tool to predict conversion from mild cognitive impairment to probable Alzheimer's disease* Alzheimers Dement, 2014. **10**(6): p. 646-55.

41. H.B. Mehta, V. Mehta, C.L. Tsai, H. Chen, R.R. Aparasu, and M.L. Johnson, *Development and Validation of the RxDx-Dementia Risk Index to Predict Dementia in Patients with Type 2 Diabetes and Hypertension* J Alzheimers Dis, 2016. **49**(2): p. 423-32.

42. T.C. Ling, C.C. Chang, C.Y. Li, J.M. Sung, C.Y. Sun, K.J. Tsai, Y.Y. Cheng, J.L. Wu, Y.T. Kuo, and Y.T. Chang, *Development and validation of the dialysis dementia risk score: A retrospective, population-based, nested case-control study* Eur J Neurol, 2022. **29**(1): p. 59-68.

43. X.-H. Hou, J. Suckling, X.-N. Shen, Y. Liu, C.-T. Zuo, Y.-Y. Huang, H.-Q. Li, H.-F. Wang, C.-C. Tan, and M. Cui, *Multipredictor risk models for predicting individual risk of Alzheimer’s disease* Journal of translational medicine, 2023. **21**(1): p. 768.

44. L.G. Exalto, C.P. Quesenberry, D. Barnes, M. Kivipelto, G.J. Biessels, and R.A. Whitmer, *Midlife risk score for the prediction of dementia four decades later* Alzheimers Dement, 2014. **10**(5): p. 562-70.

45. E.J. Chosy, S.D. Edland, N. Gross, M.J. Meyer, C.Y. Liu, L.J. Launer, and L.R. White, *The CAIDE Dementia Risk Score and the Honolulu-Asia Aging Study* Dement Geriatr Cogn Disord, 2019. **48**(3-4): p. 164-171.

46. B.C.M. Stephan, E. Pakpahan, M. Siervo, S. Licher, G. Muniz-Terrera, D. Mohan, D. Acosta, G. Rodriguez Pichardo, A.L. Sosa, I. Acosta, J.J. Llibre-Rodriguez, M. Prince, L. Robinson, and M. Prina, *Prediction of dementia risk in low-income and middle-income countries (the 10/66 Study): an independent external validation of existing models* Lancet Glob Health, 2020. **8**(4): p. e524-e535.

47. M.H. Huque, S. Kootar, R. Eramudugolla, S.D. Han, M.C. Carlson, O.L. Lopez, D.A. Bennett, R. Peters, and K.J. Anstey, *CogDrisk, ANU-ADRI, CAIDE, and LIBRA Risk Scores for Estimating Dementia Risk* JAMA Netw Open, 2023. **6**(8): p. e2331460.

48. K.J. Anstey, N. Cherbuin, P.M. Herath, C. Qiu, L.H. Kuller, O.L. Lopez, R.S. Wilson, and L. Fratiglioni, *A self-report risk index to predict occurrence of dementia in three independent cohorts of older adults: the ANU-ADRI* PLoS One, 2014. **9**(1): p. e86141.

49. S. Kootar, M.H. Huque, R. Eramudugolla, D. Rizzuto, M.C. Carlson, M.C. Odden, O.L. Lopez, C. Qiu, L. Fratiglioni, S.D. Han, D.A. Bennett, R. Peters, and K.J. Anstey, *Validation of the CogDrisk Instrument as Predictive of Dementia in Four General Community-Dwelling Populations* The Journal of Prevention of Alzheimer's Disease, 2023. **10**(3): p. 478-487.

50. S.J.B. Vos, M.P.J. van Boxtel, O.J.G. Schiepers, K. Deckers, M. de Vugt, I. Carriere, J.F. Dartigues, K. Peres, S. Artero, K. Ritchie, L. Galluzzo, E. Scafato, G.B. Frisoni, M. Huisman, H.C. Comijs, S.F. Sacuiu, I. Skoog, K. Irving, C.A. O'Donnell, F.R.J. Verhey, P.J. Visser, and S. Kohler, *Modifiable Risk Factors for Prevention of Dementia in Midlife, Late Life and the Oldest-Old: Validation of the LIBRA Index* J Alzheimers Dis, 2017. **58**(2): p. 537-547.

51. O.J.G. Schiepers, S. Kohler, K. Deckers, K. Irving, C.A. O'Donnell, M. van den Akker, F.R.J. Verhey, S.J.B. Vos, M.E. de Vugt, and M.P.J. van Boxtel, *Lifestyle for Brain Health (LIBRA): a new model for dementia prevention* Int J Geriatr Psychiatry, 2018. **33**(1): p. 167-175.

52. K. Deckers, M. Barbera, S. Kohler, T. Ngandu, M. van Boxtel, M. Rusanen, T. Laatikainen, F. Verhey, H. Soininen, M. Kivipelto, and A. Solomon, *Long-term dementia risk prediction by the LIBRA score: A 30-year follow-up of the CAIDE study* Int J Geriatr Psychiatry, 2020. **35**(2): p. 195-203.

53. J. Li, M. Ogrodnik, S. Devine, S. Auerbach, P.A. Wolf, and R. Au, *Practical risk score for 5-, 10-, and 20-year prediction of dementia in elderly persons: Framingham Heart Study* Alzheimers Dement, 2018. **14**(1): p. 35-42.
